# MicroRNA 27a-3p Regulates Antimicrobial Responses of Murine Macrophages Infected by *Mycobacterium avium* subspecies *paratuberculosis* by Targeting Interleukin-10 and TGF-β-Activated Protein Kinase 1 Binding Protein 2

**DOI:** 10.3389/fimmu.2017.01915

**Published:** 2018-01-11

**Authors:** Tariq Hussain, Deming Zhao, Syed Zahid Ali Shah, Jie Wang, Ruichao Yue, Yi Liao, Naveed Sabir, Lifeng Yang, Xiangmei Zhou

**Affiliations:** ^1^State Key Laboratories for Agrobiotechnology, Key Laboratory of Animal Epidemiology and Zoonosis, Ministry of Agriculture, National Animal Transmissible Spongiform Encephalopathy Laboratory, College of Veterinary Medicine, China Agricultural University, Beijing, China

**Keywords:** *Mycobacterium avium* subspecies *paratuberculosis*, microRNAs, miR-27a, mitogen-activated protein kinase p38, TAK1 binding protein 2, interleukin-10

## Abstract

*Mycobacterium avium* subspecies *paratuberculosis* (MAP) persistently survive and replicate in mononuclear phagocytic cells by adopting various strategies to subvert host immune response. Interleukin-10 (IL-10) upregulation *via* inhibition of macrophage bactericidal activity is a critical step for MAP survival and pathogenesis within the host cell. Mitogen-activated protein kinase p38 signaling cascade plays a crucial role in the elevation of IL-10 and progression of MAP pathogenesis. The contribution of microRNAs (miRNAs) and their influence on the activation of macrophages during MAP pathogenesis are still unclear. In the current study, we found that miRNA-27a-3p (miR-27a) expression is downregulated during MAP infection both *in vivo* and *in vitro*. Moreover, miR-27a is also downregulated in toll-like receptor 2 (TLR2)-stimulated murine macrophages (RAW264.7 and bone marrow-derived macrophage). ELISA and real-time qRT-PCR results confirm that overexpression of miR-27a inhibited MAP-induced IL-10 production in macrophages and upregulated pro-inflammatory cytokines, while miR-27a inhibitor counteracted these effects. Luciferase reporter assay results revealed that IL-10 and TGF-β-activated protein kinase 1 binding protein 2 (TAB 2) are potential targets of miR-27a. In addition, we demonstrated that miR-27a negatively regulates TAB 2 expression and diminishes TAB 2-dependent p38/JNK phosphorylation, ultimately downregulating IL-10 expression in MAP-infected macrophages. Furthermore, overexpression of miR-27a significantly inhibited the intracellular survival of MAP in infected macrophages. Our data show that miR-27a augments antimicrobial activities of macrophages and inhibits the expression of IL-10, demonstrating that miR-27a regulates protective innate immune responses during MAP infection and can be exploited as a novel therapeutic target in the control of intracellular pathogens, including paratuberculosis.

## Introduction

Paratuberculosis or Johne’s disease is characterized by chronic granulomatous enteritis predominantly observed in ruminants. It is caused by an obligate non-tuberculous *Mycobacterium avium* subspecies *paratuberculosis* (MAP), a member of *M. avium* complex. Paratuberculosis has a global distribution and is listed under the World Organization for Animal Health (OIE) *Terrestrial Animal Health Code*. Studies demonstrated that more than 50% of dairy cattle herds were positive for MAP antibodies in Europe and USA (1, 2). In addition, the USDA reports suggested an increasing trend of MAP infections in US dairy herds, from 21.6% in 1996 to 91.1% in 2007 (3). A recent study from northeastern part of China reported that the prevalence of paratuberculosis is 4.8% at the animal level and 50.0% at the herd level (4). Paratuberculosis inflicts heavy economic losses to the dairy industry in terms of weight loss, reduced milk production, infertility, culling of young animals, and the treatment of secondary diseases (2). Animals of all ages are susceptible to paratuberculosis, and the infection is acquired by fecal–oral route or ingestion of contaminated milk in young animals (5, 6). MAP infection poses a serious threat to human population apart from affecting a range of animal species. Contaminated food or water is the major source of MAP infection in humans (7). The relation between MAP and *Morbus Crohn’s* disease (CD) of humans was reported for the first time by Dalziel in 1913 (8). Various studies have documented the involvement of MAP in CD, but the causation of CD by MAP has not been confirmed (7, 9). In light of the current knowledge about MAP and its relationship with human disease, the majority of researchers support the theory that MAP causes CD in some genetically susceptible human hosts, but additional proof is needed for the confirmation of MAP as a causative agent of CD (10).

Macrophages are important cells in the regulation of protective immune responses for the elimination of intracellular pathogens. On the other hand, they are the key cells involved in the pathogenesis of paratuberculosis by providing a suitable intracellular environment for the persistent survival and growth of MAP (11). Pattern recognition receptors (PRRs) are crucial in the phagocytosis of mycobacterium by mononuclear phagocytic cells such as macrophages (12). Toll-like receptors (TLRs) are a group of important PRRs involved in the activation of various signaling pathways in macrophages during mycobacterium infection (13). It has been reported that the signaling cascades initiated by TLR2 are important for the pathogenesis of mycobacterium *via* inhibition of antimicrobial responses in macrophages (14). Mitogen-activated protein kinase p38 (MAPK-p38) signaling pathway in bovine mononuclear phagocytic cells is activated by MAP *via* TLR2, resulting in overexpression of interleukin (IL)-10 and downregulation of IL-12 (15, 16). In addition, the binding of antigens to TLR2/4 and CD14 receptors of phagocytic cells induces an increased production of IL-10, while inhibiting the development of protective immune responses, ultimately promoting the survival and growth of intracellular pathogens (17).

Cytokines are a diverse group of secretory proteins playing an important role in intercellular signaling mechanisms for the generation of immune responses in various diseases. As a result of the use of immunomodulatory drugs, some cytokines inhibit the production of other cytokines, either by inverse-regulatory function or sharing a common signaling pathway in similar types of cells (18). IL-10 is one of the potent inhibitory cytokines that inversely regulate the expression of various pro-inflammatory mediators induced by TLR signaling cascades in immune-modulating cells (19). IL-10 also plays an important role in repairing tissue injury, but on the other hand it is involved in the persistent survival of mycobacterium in macrophages (20). IL-10 promotes the ability of *Mycobacterium tuberculosis* (M.tb) to evade immune responses and mediate long-term pathogenesis in the lungs (21). Furthermore, anti-IL-10 antibodies treatment increased immunological balance and controlled M.tb burden and pathogenesis in monkeys (22). In addition, IL-10 production is highly specific to MAP infection in comparison to other Mycobacterium species, suggesting that IL-10 detection can be used as a diagnostic tool in subclinical paratuberculosis (20). Overall, IL-10 promotes anti-inflammatory genes and is associated with a dampening of the protective immune responses (23).

Mitogen-activated protein kinase p38 signaling pathway is implicated as an emerging signaling pathway involved in the pathogenesis of MAP in mononuclear phagocytic cells (24, 25). MAPK-p38 signaling can be pro-apoptotic or anti-apoptotic, depending on the cell type and stimulus strength (26). MAP infection leads to the activation of MAPK-p38 pathway by altering antibacterial activity of macrophages and promoting IL-10 production required for the survival of MAP in infected macrophages (15, 27). Furthermore, a set of six recombinant proteins of MAP promote MAPK-p38 phosphorylation and strongly induce IL-10 transcription in bovine monocyte-derived macrophages (16).

MicroRNAs (miRNAs) are small non-coding endogenous RNA fragments, 21–25 nucleotides in length, regulating gene expression by binding at the 3′-UTR of the targeted mRNA genes (28, 29). Recent studies have determined the role of miRNAs as a key mediator of the host immune response to infection, mostly by regulating proteins involved in innate and adaptive immune pathways (30). In addition, miRNAs are involved in macrophage polarization and T cell and B cell differentiation (31, 32). The miR-24 and miR-27a modulate immune response by inhibiting Th2 regulation through targeting IL-4 and GATA binding protein 3 (GATA3) of mouse CD4 T cells (33). Early studies reported that miR-27a and miR-27b have antiviral activities against murine cytomegalovirus infection in different mouse cell lines (34). Ji and colleagues determined that both miR-27a and miR-27b target the 3′-UTR of the retinoid X receptor α and play a similar role in regulating fat metabolism and cell proliferation during rat hepatic stellate cell activation (35). In addition, Tak et al. reported that miR-27a/b similarly promotes mitochondrial membrane potential and mitochondrial ATP level by targeting the 3′-UTR of the mitochondrial fission factor (36). Recent studies reported the profile of miRNA’s in MAP-infected animals that can be considered as an emerging strategy for the control of paratuberculosis. These miRNAs maintain their integrity during long-term storage and are considered an effective diagnostic tool (37, 38). However, the regulatory mechanism of innate immunity in paratuberculosis by miRNAs is unclear. IL-10 is an important mediator to suppress the early innate immune responses against paratuberculosis (20). Recent studies have demonstrated that miR-27a participates in the inflammatory responses of macrophages stimulated by LPS (39). In addition, miR-27a is one of the differentially regulating miRNAs in U937 macrophages activated with M.tb heat shock protein Hsp16.3 (40).

**Figure 1:**
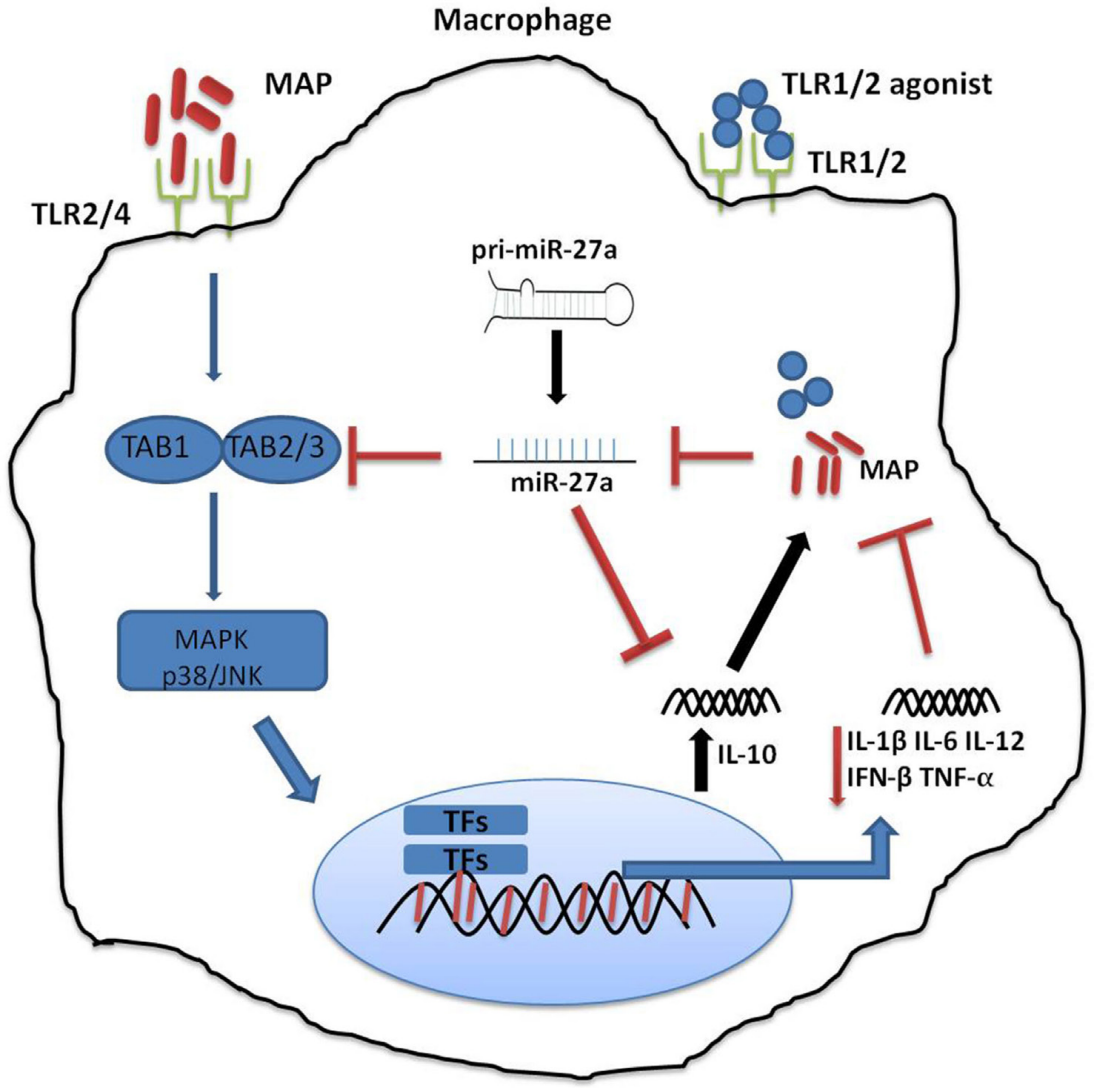
Schematic diagram depicting the role of miR-27a in *Mycobacterium avium* subspecies *paratuberculosis* (MAP)-mediated signaling pathways in macrophage. Mitogen-activated protein kinase (MAPK) pathway is triggered by toll-like receptors (TLRs), and TLR2/4 is more critical in signaling through various adopter proteins. A complex of protein integrations are involved in the downsignaling cascades consisting of TGF-β-activated protein kinase 1 (TAK1), TAK binding protein 1 (TAB 1), and TAB 2 or TAB 3. This interaction induces phosphorylation of TAK1, which results in the activation of the MAPK-p38 (mitogen-associated protein kinases). This leads to the activation and nuclear translocation of transcription factors, which play a critical role in the expression of pro-inflammatory and anti-inflammatory cytokines such as interleukin (IL)-10. MAP predominantly induces an immunosuppressive cytokine, IL-10, which subverts protective immune responses and promotes MAP growth and survival. MAP downregulates the expression of miR-27a, while miR27a inhibits the activation of the MAPK-p38 signaling pathway by targeting TAB 2. Furthermore, miR-27a negatively regulates the expression of IL-10 by directly targeting IL-10 mRNA at its 3′-UTR.

The main objective of our study was to evaluate the effect of miR-27a on the production of IL-10, the IL-10 regulatory pathway—especially MAPK-p38—and macrophages antimicrobial response for the control of MAP pathogenesis. We observed a gradual decrease in the level of miR-27a in MAP-infected murine macrophages and in the spleen and intestinal tissues of MAP-challenged mice. Overexpression of miR-27a decreased the production of IL-10 in MAP-infected macrophages. Also, miR-27a significantly inhibited the expression of TGF-β-activated protein kinase 1 (TAK1) binding protein 2 (TAB 2)/3, an important component of the MAPK signaling pathway (Figure 1). Upregulation of miR-27a diminished TAB 2-dependent p38/JNK phosphorylation, subsequently downregulated IL-10 in MAP-infected macrophages. Our results provide a novel role for miR-27a as a potential biomarker that can be exploited for control strategies of MAP infection.

## Materials and Methods

### Ethics Statement

Animal experiments were performed according to the protocols for the care of laboratory animals, Ministry of Science and Technology People’s Republic of China and approved animal care and use committee (IACUC) protocols at China Agricultural University of Beijing (20110611-01). All other experiments were carried out in accordance with the University Institutional Biosafety Committee approved protocol number 20110611-0.

### Reagents

MicroRNA miR-27a negative control, mimic, inhibitor control, and inhibitor were purchased from Shanghai GenePharma. Pam3Cys-Ser-(Lys)4 (ab142085), a TLR1/TLR2 agonist, was purchased from Abcam Biotechnology; lipofectamine 2000 from Invitrogen Thermo Fisher Scientific; and BBL Middlebrook OADC (211886) enrichment and mycobactin J from Becton, Dickinson and Sparks Company (USA), respectively. Macrophage colony-stimulating factor (M-CSF) was purchased from Peprotech Technology and total RNA extraction kit (ca. RN2802) from Aidlab Biotechnologies. The miR-27a (CD202-0033) and internal control S5 (CD202-0012) primers were from TIANGEN BIOTECH (Beijing). Primers for IL-1β, IL-6, IL-10, IL-12, interferon (IFN)-β, TNF-α, and GAPDH were purchased from Genewiz Technology. The calorimetric mouse IL-10, TNF-α, and IL-6 ELISA kit (KE10008, KE10007, and KE10002) were from Proteintech. Mouse IL-12 ELISA kit (ca. CSB-E07360m) was from Cusabio Co. Ltd. Rabbit polyclonal anti-p38 MAPK antibody (9211), rabbit polyclonal anti-phospho-p38 antibody (9211), rabbit monoclonal anti-phospho-JNK antibody (4668T), and rabbit monoclonal anti-phospho-ERK antibody (4370) were purchased from Cell Signaling Technology. Rabbit polyclonal anti-TAB 2 (MAP3K7IP2) polyclonal antibody (14410-1-Ap), rabbit polyclonal anti-GAPDH antibody (10494-1-AP), and goat anti-rabbit IgG (heavy and light chains) peroxidase-conjugated secondary antibody (SA000012) were purchased from Proteintech Biotechnology. Rabbit polyclonal anti-MAP3K7IP3 (TAB 3) antibody (bs5424R) was from Beijing Biosynthesis Biotechnology. Plasmid of mRNA for IL-10 and TAB 2 wild and mutant were constructed by Synbio Technology. Dual luciferase Reporter Assay kit (RFE: E1910, 205415) from Promega technology, USA. CellTiter 96Aqueous one solution cell proliferation assay kit (ca. G3580, 0000180214) from Promega technology, USA.

### Preparation of Bacterial Culture

We used two strains of MAP (MAP-0908 and MAP k-10). MAP-0908 was isolated from a naturally infected cattle farm in the Shandong province of China. Molecular characterization of the MAP-0908 strain was done at the Transmissible Spongiform Encephalopathy Diagnostic Laboratory (China Agricultural University Beijing). Organisms were confirmed as MAP by cultural characteristics, while further species-specific DNA sequences by a standard PCR were performed for molecular characterization (41). K-10 strain of MAP 35716 was a kind gift from Prof. Paul Barrow, from the University of Nottingham at Leicestershire, UK. A stock culture of both strains of MAP was maintained in Middlebrook 7H9 medium supplemented with 1% glycerol, 10% oleic-acid-dextrose-catalase (OADC), and mycobactin J (2.0 mg/l) (Allied Monitor, Fayette, MO, USA) at 37°C. The organisms were grown to a concentration of 10^8^/ml for 2–3 weeks before being used for cell infection.

### Isolation of Bone Marrow-Derived Macrophages (BMDMs)

We used murine macrophages (RAW264.7 and BMDM) for our experiments. It has been demonstrated that MAP-infected RAW264.7 cells and sheep blood monocyte-derived macrophages show similar expression of TLR2 receptors (42). Furthermore, RAW264.7 cells are more differentiated than THP-1 cells, thus having higher phagocytic ability (43). Mouse bone marrow-derived macrophages were prepared and cultured as described previously (44). In brief, BMDMs were prepared from cells obtained from femurs, tibia, and humerus of 6- to 8-week-old C57BL/6J mice and cultured in RPMI supplemented with 10% heat-inactivated FBS (Hyclone) in the presence of 10 ng/ml of M-CSF (Peprotech) or GM-CSF (Peprotech) and 1% Penicillin-Streptomycin. At day four, non-adherent cells were collected and cultured for three more days in the presence of fresh RPMI containing M-CSF (10 ng/ml). These primary macrophages were cultured for 7–10 days in cell culture flasks. After that, the adherent BMDM cells (6–8 × 10^6^ cells per dish) were collected and plated in 12-well cell culture plates for further experiments (45). RAW264.7 macrophages were taken from cold storage (−80°C) and cultured in cell culture flask in DMEM supplemented with 10% FBS and 1% penicillin–streptomycin for 3 days. The cells were transferred to 12-well cell culture plates 12–18 h before transfection.

### Cells Transfection and Infection

Cells were allowed to attach overnight in 12-well plates (2 × 10^5^ cells in each well). The following day, miR-27a mimic, inhibitor, and control were individually transfected into RAW264.7 and BMDM cells using lipofectamine 2000 reagent, according to manufacturer’s instructions. After 6 h, the original medium was replaced with fresh medium supplemented with 10% FBS (46). After 48 h post-transfection, macrophages were infected at a multiplicity of infection (MOI) 20:1 (bacteria/cell) with live MAP in cell culture medium without antibiotic (47). Following incubation for 3 h at 37°C in 5% CO_2_, the supernatant was discarded and each well was washed three times with sterile phosphate-buffered saline (PBS) to remove non-adherent MAP bacilli. After washing, fresh DMEM medium supplemented with 10% serum was added for the specified time period. The cells were harvested 6 and 18 h postinfection for analysis of total protein, total RNA, and cell supernatant. All samples were stored at −80°C until further use.

### Quantitative Real-time PCR

Total RNA (including miRNA) was extracted from RAW264.7 and BMDM cells with TRIzol reagent (Invitrogen, Carlsbad, CA, USA) according to the manufacturer’s guidelines. To evaluate the expression levels of miRNAs and mRNAs, reverse transcription was done by using miRcute Plus miRNA First strand cDNA synthesis kits (obtained from Thermo Scientific and TIANGEN BIOTECH Beijing, China). To construct cDNA we used Thermo Scientific RevertAid First Strand cDNA synthesis kit according to the manufacturer’s instructions. Quantitative real-time PCR was performed by means of the miRcute miRNA qPCR detection kit (SYBR Green) for miRNA’s [miR-27a and internal references, small nucleolar S5 (mouse)]. Sybr Green Master Mix Kit (Vazyme Biotech Co., Ltd.) was used for the amplification of mRNA’s genes, including cytokines, by using the 700 Fast Real-Time PCR Systems (ViiA7 Real-time PCR, ABI). GAPDH was selected as housekeeping gene for the normalization of various cytokine genes because it is considered one of the most stable endogenous controls (48).

To normalize the expression of miRNAs, Ct values were obtained to calculate fold change for miRNA. A universal miR qPCR primer (possessing the binding site with universal adaptor primer, included in the kit), miRNA-27a primer, and 5S primer as internal reference (sequence complementary to the miRNA) were used to complete the real-time PCR reaction. miRNA qRT-PCR cycle parameters were as follows: 94°C for 2 min, followed by 45 cycles at 94°C for 20 s and 60°C for 34 s. The expression of mRNAs for various cytokines was determined by conducting qRT-PCR as described before (49). All the primers used in the present study for cytokines detection are mentioned in Table 1. To normalize the expression level of miRNAs and mRNAs, internal controls of S5 and GAPDH were used. The relative expression levels of miRNAs and mRNAs were determined as fold change, ΔCt values were obtained as follows: ΔCt = Ct of miRNAs or mRNAs − Ct of small nucleolar RNA S5or GAPDH. ΔΔCt values were obtained as follows: ΔΔCt = ΔCt of treated groups − ΔCt of untreated control groups. Fold change was calculated by the 2^−ΔΔCt^ method (50, 51).

**Table 1:** Primers used in this study.

Primer name and sequence (5′–3′)	
Interleukin (IL)-1β (forward)	5-AAGGAGAACCAAGCAACGACAAAATA-3
IL-1β (reverse)	5-TTTCCATCTTCTTCTTTGGGTATTGC-3
IL-6 (forward)	5-CCCAATTTCCAATGCTCTCCTA-3
IL-6 (reverse)	5-AGGAATGTCCACAAACTGATATGCT-3
IL-10 (forward)	5-AGCATTTGAATTCCCTGGGTGA-3
IL-10 (reverse)	5-CCTGCTCCACTGCCTTGCTCTT-3
IL-12 (forward)	5-CCAAATTACTCCGGACGGTTCAC-3
IL-12 (reverse)	5-CAGACAGAGACGCCATTCCACAT-3
TNF-α (forward)	5-AGAGCTACAAGAGGATCACCAGCAG-3
TNF-α (reverse)	5-TCAGATTTACGGGTCAACTTCACAT-3
Interferon (IFN)-β (forward)	5-AAGAGTTACACTGCCTTTGCCATC-3
IFN-β (reverse)	5-CACTGTCTGCTGGTGGAGTTCATC-3
GAPDH (forward)	5-CGACTTCAACAGCAACTCCCACTCTTCC-3
GAPDH (reverse)	5-TGGGTGGTCCAGGGTTTCTTACTCCTT-3

### ELISA for Cytokines

The concentration of cytokines in the cells supernatant was measured by using an ELISA kit. Cells supernatant was collected at various time intervals (6 and 18 h) post-MAP (k-10 and 0908 strains) infection of different miR-27a transfected groups. All reagents, standard, and samples were prepared according to the manufacturer’s protocols. Standards and samples (100 µl each) were added into appropriate wells of 96-well ELISA plates and incubated for 2 h at 37°C in a humid environment. After incubation, the liquid was discarded and the plates washed 4–5 times with washing buffer. Then, 100 µl of detection antibody solution was added into each well, incubated in humid conditions at 37°C for 1 h, and washed as previously. Then we added 100 µl of HRP-conjugated antibodies to each well for 40 min and incubated at 37°C in a humid environment and washed again. After that, we added 100 µl of TMB substrate for 10–30 min at room temperature in the dark. To stop the reaction, we added 100 µl stop solution to each well. A standard curve was obtained using twofold dilutions of the standard for each independent experiment. To obtain the optical density (OD), plate was read at 450 nm with a wavelength correction of 630 nm by using an ELISA plate reader. The ELISA results were obtained from two independent experiments. Samples were added into triplicate wells in each independent experiment.

### Western Blot Analysis

For total protein extraction, cells were washed twice with ice-cold PBS and homogenized with RIPA buffer containing a cocktail of protease and phosphatase inhibitor (Sigma Aldrich, St. Louis, MO, USA) for 20 min on ice. Afterward, samples were sonicated for 20 s and then centrifuged at 12,000 × *g* for 20 min at 4°C. The resulting supernatants were collected and boiled for 10 min after the addition of loading buffer (250 nM Tris HCl 6.8 pH, 10% sodium dodecyl sulfate, 0.5% bromophenol blue, 50% glycerol, and 0.5 M dithiothreitol) (52). Equal amounts of protein were separated by 12% or 10% sodium dodecyl sulfate polyacrylamide gel electrophoresis and transferred onto polyvinylidene difluoride membranes (Millipore, Billerica, MA, USA). After blocking with 5% skim milk prepared in Tris buffered saline Tween-20, membranes were incubated overnight at 4°C with primary antibodies. After incubation, membranes were rinsed three times in TBST and then incubated for 60 min at 37°C with HRP-labeled secondary antibodies. Signals were detected using an enhanced chemiluminescence detection kit (Bio-Rad, USA).

### Dual-Luciferase Reporter Assay

The binding elements for miR-27a at the 3′-UTR of IL-10 and TAB 2 mRNAs were obtained by PCR amplification using mouse genomic DNA as template and cloned into pMir-Reporter Luciferase vector (Synbio Tech). A mutant form of mouse IL-10 and TAB-2 3′-UTR was cloned into a pmirGLO vector by site-directed mutagenesis using a WT clone. HEK293 and BMDM cells were cultured in 24-well plates (2.5 × 10^4^ cells/well) overnight and transfected with 50 nM control mimics or 50 nM/ml miR-27a mimics using a Lipofectamine 2000. Six hours post-transfection, cells were again transfected with 100 ng pMir-Reporter constructs of IL-10 and TAB 2 wild- and mutant type of plasmid. Twenty-four hours after transfection, cells were lysed in lysis buffer, and a luciferase assay was performed using the Dual Luciferase reporter system (Promega), according to the manufacturer’s instructions (53). The results were obtained from three independent experiments, and all samples were collected in three replicates in each experiment.

### CFU Assay

To assess bacterial viability, BMDM and RAW264.7 macrophages (2 × 10^5^ cells in each well) were cultured in 12-well plates overnight and infected with MAP (0908 and k-10, MOI is 20:1) strains for 3 h and then washed thrice with warm PBS to remove extracellular bacteria. Thereafter, the infected cells were incubated for the indicated time periods and lysed at the specified time points with 0.1% Triton X 100. Appropriate dilutions were prepared for all transfected groups and plated on Middlebrook 7H10 agar plates supplemented with mycobactin-J and OADC in triplicate. Inoculated plates were incubated at 37°C, and colonies were counted 5 weeks after plating.

### Cell Viability Assay

Cell viability was assessed by the MTS tetrazolium assay. BMDM and RAW264.7 cells were cultured in 96-well plates overnight before transfection. Cells were transfected with mimic control, mimic, inhibitor control, and inhibitor of miR-27a for 48 h. MTS reagent (20 µl) was added in each well, and then cells were incubated at 37°C for 3 h in a humidified, 5% CO_2_ atmosphere. OD was quantified by measuring the absorbance at 490 nm wavelength with an ELISA plate reader.

### Statistical Analysis

All experiments were performed on three separate occasions. Data are expressed as mean ± SD. Student’s *t*-test was used for comparison between two groups. One-way ANOVA followed by Bonferroni’s multiple comparison tests was performed for multiple-group comparisons. Statistical analysis was carried out by using GraphPad Prism 5 software (GraphPad, La Jolla, CA, USA). Densitometric analysis of digitalized images was done by using ImageJ software (National Institute of Health, Bethesda, MD, USA). *p* Values < 0.05 were considered statistically significant.

## Results

### MAP Infection Decreases miR-27a Expression in Macrophages and Mice

**Figure 2:**
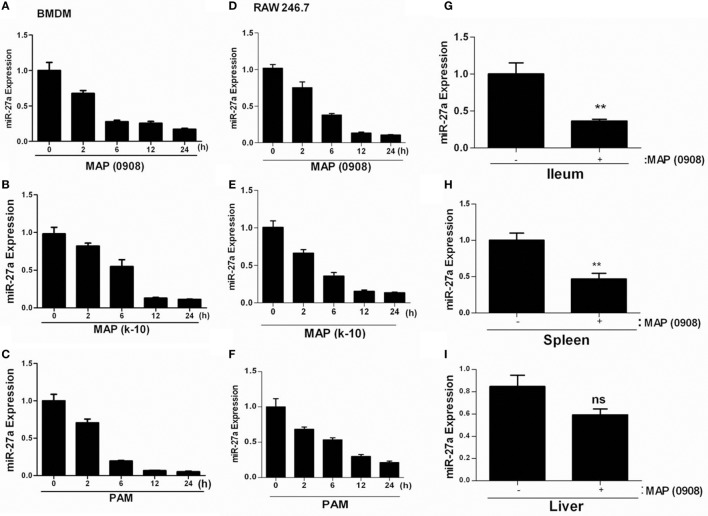
*Mycobacterium avium* subspecies *paratuberculosis* (MAP) infection decreases miR-27a expression in macrophages and mice. **(A,B)** Bone marrow-derived macrophage (BMDM) and **(D,E)** RAW264.7 cells were infected with MAP (k-10 and 0908) for the indicated time period, and miR-27a expression was subsequently evaluated by using qRT-PCR. **(C)** RAW264.7 and **(F)** BMDMs were stimulated with 1 µg/ml Pam3Cys-Ser-(Lys)4 [PAM toll-like receptor (TLR)1/2 agonist] for the indicated time period, and miR-27a expression was examined using qRT-PCR. **(G–I)** The expression levels of miR-27a were measured in the intestine (ilium) **(G)**, spleen **(H)**, and liver **(I)** of negative control or MAP (0908)-infected C57BL/6 mice by qRT-PCR analysis. All data above represent mean ± SD for three independent experiments. *p < 0.05 and **p < 0.001.

To determine whether miR-27a plays a role in the immune responses against MAP infection, we investigated the expression of miR-27a *in vivo* and *in vitro*. We infected murine BMDMs and RAW264.7 cells with two strains of MAP (MAP k-10 and MAP 0908) at MOI 20:1 (bacteria/cell) and with TLR2 agonist. To ensure maximum infection of macrophages with MAP, we conducted phagocytosis assays at different levels of MOI (5:1, 10:1, and 20:1) before establishing the dose of infectivity (Figures S1A,B in Supplementary Material). The expression of miR-27a was measured by using qRT-PCR analysis. MAP infection decreased the expression of miR-27a in a time-dependent manner in BMDM (Figures 2A,B) and RAW264.7 cells (Figures 2D,E). Additionally, TLR2 agonist also showed a decrease in the expression of miR-27a in both types of macrophages (Figures 2C,F). The minimum level of miR-27a was detected at 12–24 h time intervals in all groups. Moreover, for *in vivo* determination of miR27a we challenged mice with MAP (0908) *via* intraperitoneal route. After 4 weeks postinfection, the expression level of miR-27a was significantly downregulated in different tissues of infected mice (Figures 2G–I). Various studies have reported that TLR2 receptors play an important role in the initiation of signaling cascades in macrophages to favor the persistent survival and growth of MAP (17). Our data suggest that downregulation of miR-27a in response to MAP strains and TLR2 treatment shares a common response in both types of macrophages and indicate that the expression of miR-27a in MAP- and TLR2-treated cells may share a common signaling mechanism.

### miR-27a Attenuates the Regulation of IL-10 in Macrophages Infected with MAP

**Figure 3:**
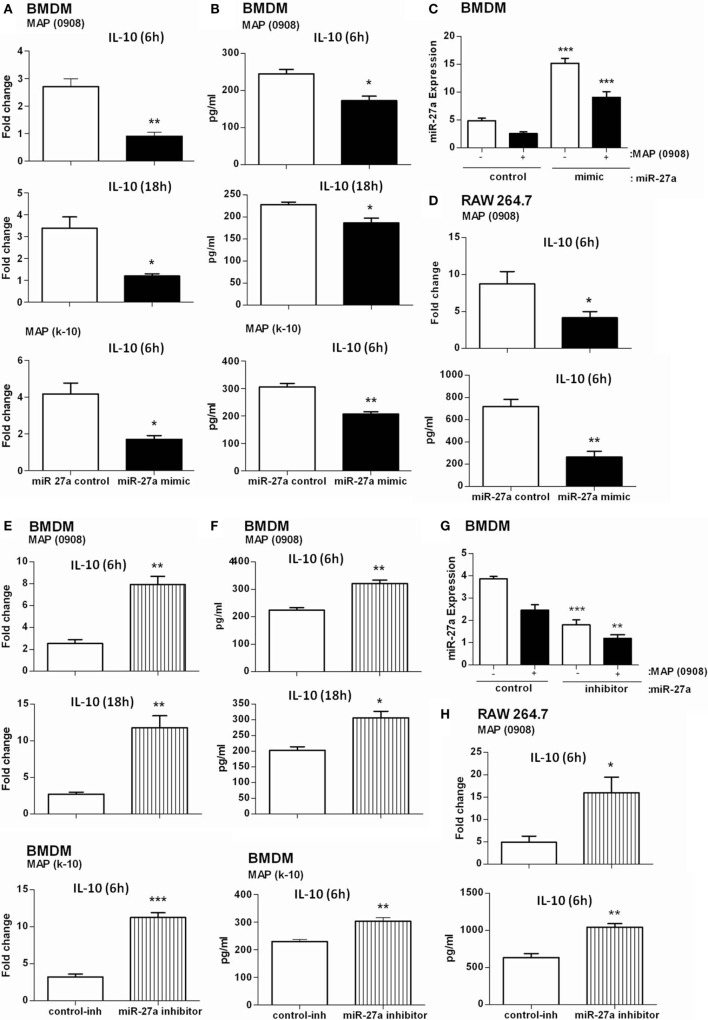
miR-27a attenuates regulation of interleukin (IL)-10 in macrophages infected by *Mycobacterium avium* subspecies *paratuberculosis* (MAP). Bone marrow-derived macrophage (BMDM) **(A,B)** and RAW264.7 **(D)** cells were transfected with 50 nM control mimics or miR-27a mimics. After 48 h, cells were infected with MAP (0908 or K-10) strain for 6 and 18 h. The mRNA and protein levels of the anti-inflammatory cytokine IL-10 were calculated by using qRT-PCR and ELISA. BMDM **(E,F)** and RAW264.7 **(H)** cells were transfected with 50 nM control inhibitors or miR-27a inhibitors. After 48 h, cells were infected with MAP (0908 or K-10) strain for 6 and 18 h. The mRNA and protein levels of the anti-inflammatory cytokine IL-10 were determined by qRT-PCR and ELISA. **(C,G)** BMDMs were transfected with miR-27a control, mimic **(C)**, or inhibitor **(G)** and then infected with MAP. The expression levels of miR-27a were determined by qRT-PCR analysis. Statistical analysis was carried out by using one-way ANOVA followed by Bonferroni’s multiple comparison tests. *p < 0.05, **p < 0.001, and ***p < 0.001.

It has been reported that TLR2-activated macrophages elevate the expression of IL-10 in response to MAP infection (20). IL-10 is one of the potent anti-inflammatory mediators that prevent tissue injury from excessive inflammatory reactions and also contribute to the pathogenesis of MAP (15, 54). More recently, it has been reported that miR-27a controls various targets to regulate the immune system (34, 39). We asked whether miR-27a participates in inflammatory responses associated with MAP (0908 and k-10) infection. We found that the level of miR-27a in BMDM cells increased by transfecting the cells with miR-27a mimic. We also found that upregulation of miR-27a reduced MAP-induced IL-10 expression at both 6 and 18 h postinfection (Figure 3A) and simultaneously reduced L-10 protein levels in BMDM cells after infection with MAP (Figure 3B). A similar IL-10 expression was recorded in RAW264.7 macrophages treated with miR-27a at both protein and mRNA levels (Figure 3D). In contrast, an upregulation of IL-10 was observed at protein and mRNA levels in both BMDM and RAW264.7 macrophages transfected with miR-27a inhibitor (Figures 3E,F,H). To check the transfection efficiency, BMDM cells were transfected with miR-27a control, mimic, and inhibitor for 48 h and then the expression of miR-27a observed with or without MAP infection (0908). The expression level of miR-27a was significantly higher in MAP-infected groups than in the MAP non-infected mimic-treated group (Figure 3C). In addition, miR-27a was downregulated in inhibitor treatment of both MAP-infected and MAP non-infected groups (Figure 3G). These data suggest that miR-27a negatively regulates the expression of IL-10 at both mRNA and protein levels in MAP-infected macrophages. We further analyzed the effects of miR-27a transfection on macrophage viability. BMDM and RAW264.7 cells were transfected with miR-27a (control, mimic, control-inhibitor, and inhibitor) for 48 h, and then cell viability was assessed by using the MTS assay (see Materials and Methods). Cell viability among all transfected groups showed no significant difference after miR-27a transfection (Figures S4A,B in Supplementary Material).

### miR-27a Upregulation Improves Macrophage Activation in Response to MAP Infection

**Figure 4:**
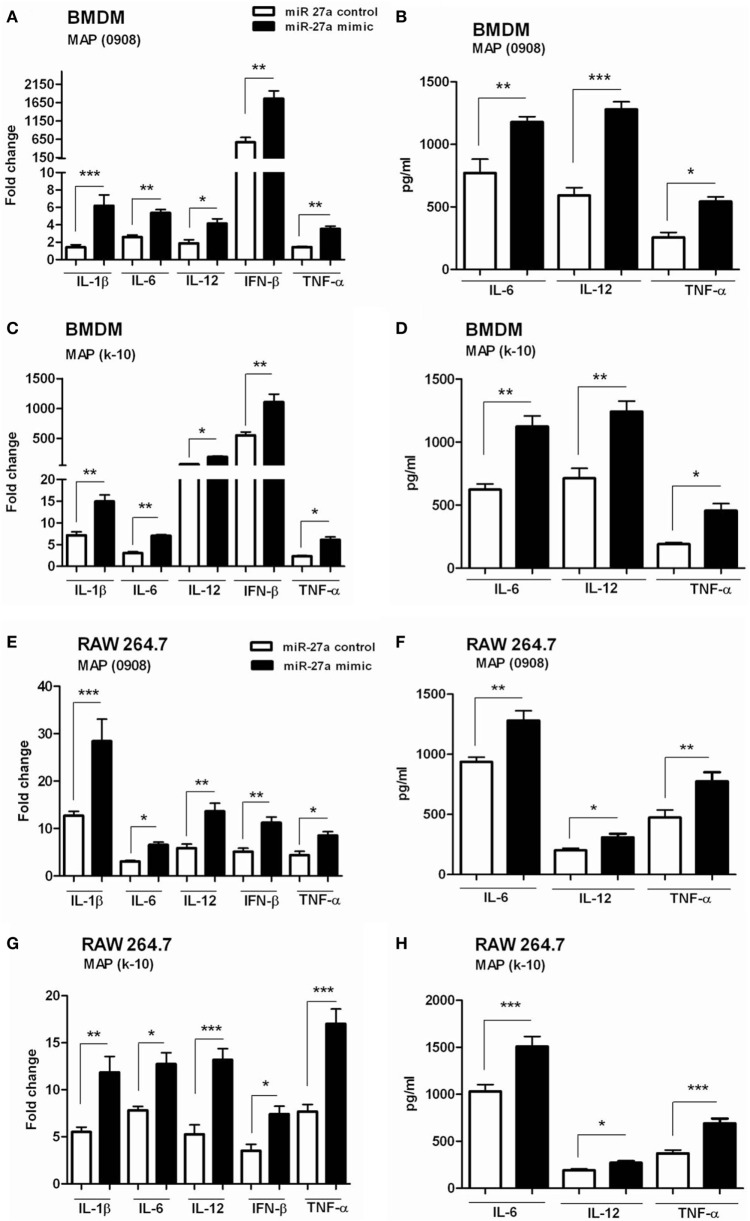
miR-27a upregulation improves macrophage activation in response to *Mycobacterium avium* subspecies *paratuberculosis* (MAP) infection. **(A–D)** Bone marrow-derived macrophage (BMDM) cells were transfected with 50 nM miR-27a control or miR-27a mimic. After 48 h, cells were infected by MAP (0908 or k-10) strain for 6 h. The mRNA and protein levels of pro-inflammatory cytokines interleukin (IL)-1β, IL-6, IL-12, interferon (IFN)-β, and TNF-α were determined by qRT-PCR **(A,C)**, and IL-6, IL-12, and TNF-α were determined by ELISA **(B,D)**. **(E–H)** RAW264.7 cells were transfected with 50 nM miR-27a control or miR-27a mimic. After 48 h, cells were infected by MAP (0908 or k-10) strain for 6 h. The mRNA and protein levels of pro-inflammatory cytokines IL-1β, IL-6, IL-12, IFN-β, and TNF-α were determined by qRT-PCR **(E,G)**, and IL-6, IL-12, and TNF-α were determined by ELISA **(F,H)**. Data represent mean ± SD from three independent experiments. *p < 0.05, **p < 0.001, and ***p < 0.001.

We have already shown that miR-27a is downregulated in MAP-infected macrophages and also observed the inhibitory effects of miR-27a on IL-10 expression. Previous studies reported that MAP inhibit the activation of macrophages by upregulating the expression of IL-10 while downregulating various pro-inflammatory mediators (15). The downregulation of miR-27a in MAP infection suggested that miR-27a could participate in the activation of macrophages. To test this hypothesis, we investigated whether miR27a participates in the expression of inflammatory mediators during MAP infection. First, we increased the level of miR-27a in BMDM cells by transfecting with miR-27a mimics for 48 h, and then cells were infected with MAP 0908 strain. After 6 h postinfection, we found a high level of pro-inflammatory cytokines, including IL-1β, IL-6, IL-12, IFN-β, and TNF-α in BMDM at both translational and transcriptional levels (Figures 4A,B). Similar results were observed for k-10 strain of MAP in infected macrophages (Figures 4C,D). Consistent with the findings in BMDM cells, upregulation of miR-27a also enhanced MAP-induced expression of IL-1β, IL-6, IL-12, IFN-β, and TNF-α in RAW264.7 cells (Figures 4E–H). In addition, we found that miR-27a also upregulates the expression of pro-inflammatory cytokines after 18 h of MAP infection, compared to miR-27a control (Figure S2 in Supplementary Material). These data suggest that miR-27a is a positive regulator for the induction of inflammatory cytokines in macrophages infected with MAP.

### Downregulation of miR-27a Inhibits Inflammatory Responses of MAP-Infected Macrophages

**Figure 5:**
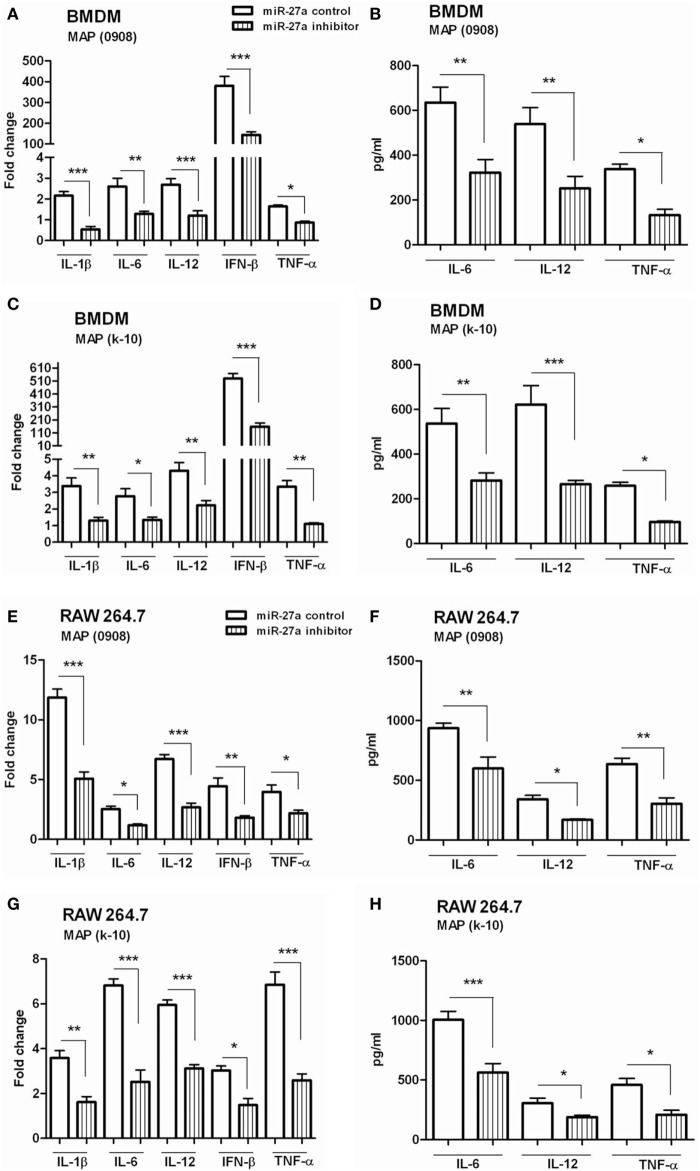
Downregulation of miR-27a inhibits inflammatory responses of *Mycobacterium avium* subspecies *paratuberculosis* (MAP)-infected macrophages. **(A–D)** Bone marrow-derived macrophage (BMDM) cells were transfected with 50 nM control inhibitors or miR-27a inhibitors. After 48 h, cells were infected by MAP (0908 or k-10) strain for 6 and 18 h. The mRNA and protein levels of pro-inflammatory cytokines interleukin (IL)-1β, IL-6, IL-12, interferon (IFN)-β, and TNF-α were determined by qRT-PCR **(A,C)**, and IL-6, IL-12, and TNF-α were determined by ELISA **(B,D)**. **(E–H)** RAW264.7 cells were transfected with 50 nM control inhibitors or miR-27a inhibitors. After 48 h, cells were infected by MAP (0908 or k-10) strain for 6 and 18 h. The mRNA and protein levels of pro-inflammatory cytokines IL-1β, IL-6, IL-12, IFN-β, and TNF-α were determined by **(E,G)** qRT-PCR, and IL-6, IL-12, and TNF-α were determined by **(F,H)** ELISA. Similar results were observed in three independent experiments. *p < 0.05, **p < 0.01, and ***p < 0.001.

We observed that mimic treatment of miR-27a promoted inflammatory responses of macrophages infected with MAP and then asked whether downregulation of miR-27a will affect macrophage response against MAP. The level of miR-27a decreased in BMDM cells by transfecting the cells with miR-27a inhibitor for 48 h and then infected them with MAP 0908. After 6 h of infection, we found significantly decreased levels of pro-inflammatory cytokines, including IL-1β, IL-6, IL-12, IFN-β, and TNF-α induced by MAP 0908 in BMDM at both translational and transcriptional levels (Figures 5A,B). Similar findings were observed for MAP k-10-infected macrophages (Figures 5C,D). In addition, the downregulation of miR-27a also inhibited various cytokines expression in RAW264.7 macrophages after infection with MAP 0908 and k-10 strains (Figures 5E–H). Furthermore, we found that downregulation of miR-27a also inhibited the expression of pro-inflammatory cytokines after 18 h of MAP infection (Figure S3 in Supplementary Material). These findings suggest that miR-27a regulates macrophage activation by abrogating the anti-inflammatory environment created by MAP in infected macrophages for prolonged survival and growth.

### miR-27a Modulates MAPK Signaling Cascade by Targeting TAB 2 and TAB 3 in MAP-Infected Macrophages

**Figure 6:**
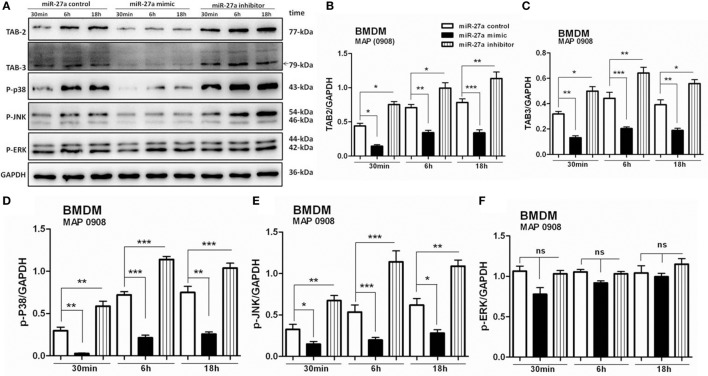
miR-27a modulates mitogen-activated protein kinase (MAPK) signaling cascades by targeting TGF-β-activated protein kinase 1 binding protein 2 (TAB 2) and TAB 3 in *Mycobacterium avium* subspecies *paratuberculosis* (MAP)-infected macrophages. **(A)** Bone marrow-derived macrophage (BMDM) cells were transfected with 50 nM miR-27a control, mimic, and inhibitor for 48 h and then infected with MAP (0908) at multiplicity of infection (MOI) of 20:1 (bacteria/cell) for 30 min, 6 h, and 18 h. The expression levels of TAB 2, TAB 3, p-P38, p-JNK, and p-ERK were detected by Western blot. **(B–F)** Western blot analysis of protein levels of TAB 2 **(B)**, TAB 3 **(C)**, p-P38 **(D)**, p-JNK **(E)** s, and p-ERK **(F)** normalized to GAPDH after transfection with miR-27a control, mimic, and inhibitor for the indicated times. Data represent mean ± SD from three independent experiments. *p < 0.05, **p < 0.001, and ***p < 0.001.

Complex signaling cascades are triggered by MAP after interaction with TLR2/4 for the activation of MAPK-p38 pathway (15). TAB 1, TAB 2, and TAB 3 are involved in the activation of TAK1 (55). Various kinases are activated by downstream signaling of TAK1 including MAPKs. We investigated whether miR-27a participates in the regulation of signaling cascades of MAPK pathway in MAP-infected macrophages. BMDM cells were transfected with miR-27a control, mimic, and inhibitor. Forty-eight hours after transfection, cells were infected with MAP 0908 strain, and the expression of various proteins of the MAPK pathway was determined by immunoblot assays. As shown in Figure 6A, upregulation of miR-27a diminished MAP (0908) induced TAB 2 (Figure 6B) and TAB 3 (Figure 6C) expression, while downregulation of miR-27a counteracted these effects. Furthermore, we examined whether miR-27a affects the components of MAPK pathway that regulate the expression of IL-10 during MAP infection. We found that upregulation of miR-27a reduced the expression of phosphorylated p38 (Figure 6D) and p-JNK (Figure 6E), while downregulation of miR-27a significantly promoted the expression of p-p38 and p-JNK at all time intervals. However, no significant difference was observed in p-ERK (Figure 6F) in BMDM cells at various time intervals among all treated groups after MAP (0908) infection. These findings suggest that miR-27a modulates MAPK signaling pathways activated by MAP in infected macrophages.

### IL-10 and TAB 2 Are Direct Targets of miR-27a

**Figure 7:**
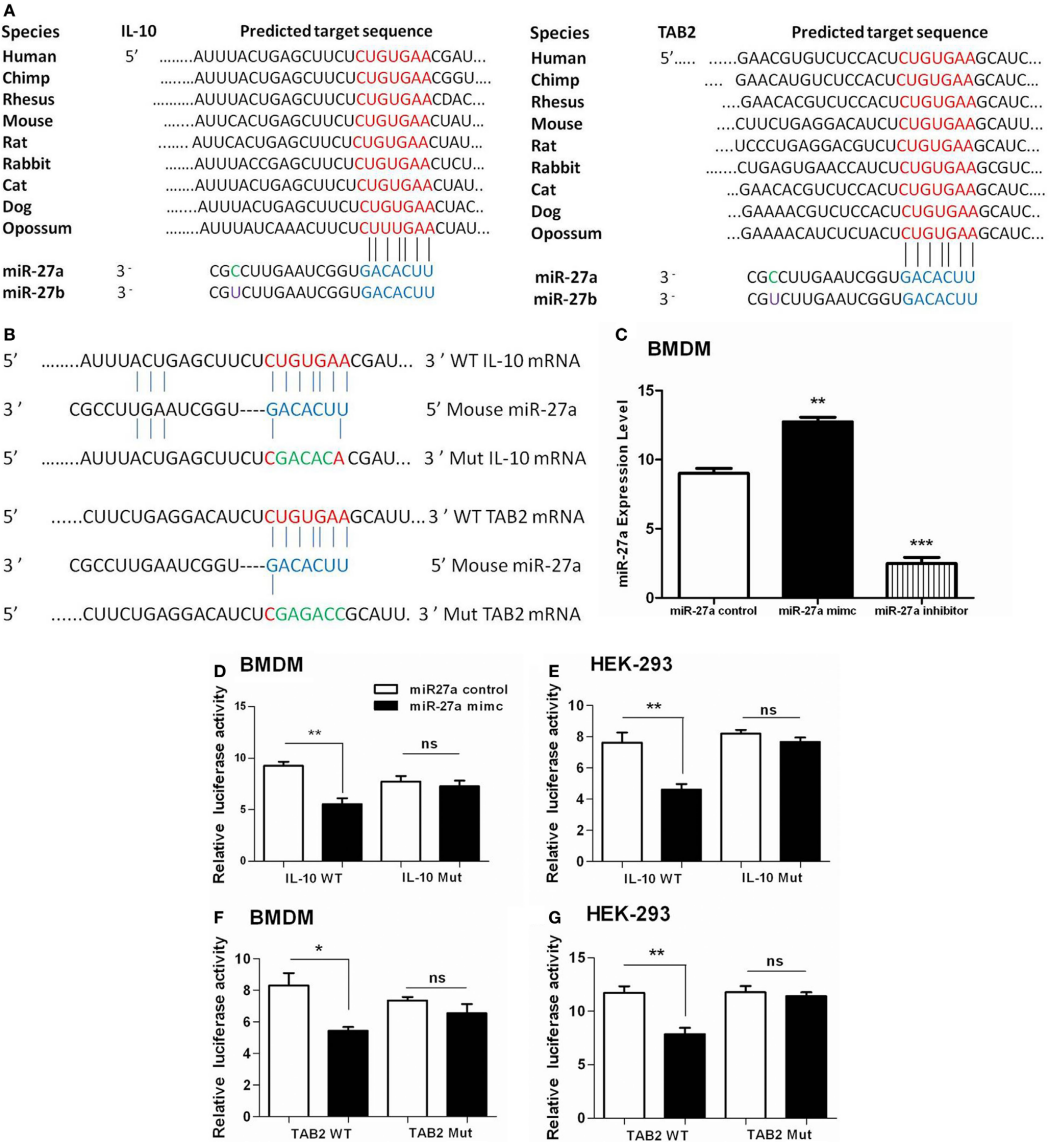
Interleukin (IL)-10 and TGF-β-activated protein kinase 1 binding protein 2 (TAB 2) are direct targets of miR-27a. **(A)** The mature sequences of miR-27a/b and the conserved sequences of the 3′-UTR of IL-10 and TAB 2 from various species are illustrated. The seed sites of miR-27a/b and its binding sites at the 3′-UTR of IL-10 and TAB 2 are shown in *blue* and *red* color, respectively. **(B)** Schematic illustration of the miR-27a targeting site at 3′-UTR of the mouse IL-10 and TAB 2 gene. The created mutation is represented in green color. **(C)** Bone marrow-derived macrophage (BMDM) cells were transfected with 50 nM miR-27a control, mimic, and inhibitor. Forty-eight hours after transfection, cells were lysed and the expression levels of miR-27a were determined by real-time PCR assay. Data represent mean ± SD from two independent experiments. **(D)** BMDM cells were transfected with 50 nM miR-27a control, miR-27a mimic and a wild-type (IL-10 WT) or mutant IL-10 3′-UTR (IL-10 Mut), and **(F)** wild-type (TAB 2 WT) or mutant TAB 2 3′-UTR (TAB 2 Mut) luciferase reporter plasmid. Luciferase activities of the BMDM cells were assessed 24 h after transfection. **(E)** HEK-293 cells were transfected with 50 nM miR-27a control, miR-27a mimic and a wild-type (IL-10 WT) or mutant IL-10 3′-UTR (IL-10 Mut) and **(G)** wild-type (TAB 2 WT) or mutant TAB 2 3′-UTR (TAB 2 Mut) luciferase reporter plasmid. Luciferase activities of the HEK-293 cells were assessed 24 h after transfection. Similar results were observed in three independent experiments. *p < 0.05, **p < 0.01, and ***p < 0.001.

To clarify whether miR-27a targets IL-10 alone or IL-10 and TAB 2 together at their 3′-UTR region, we used TargetScan and miRanda algorithm to predict miRNA targets as previously described (56, 57). We identified a putative binding site of miR-27a/b at the 3′-UTR of IL-10 and TAB 2 transcript in mice and also in other species (Figure 7A). Luciferase reporter vectors were constructed for wild and mutant 3′-UTR for IL-10 and TAB 2 target site of miR-27a (Figure 7B). We found that upregulation of miR-27a reduced the activity of the luciferase reporter in BMDM and HEK293 cells containing IL-10 wild-type reporter, while miR-27a failed to inhibit luciferase activity in cells having IL-10 mutant-type reporter (Figures 7D,E). We also found that miR-27a decreased the activity of the luciferase reporter that contained the wild-type 3′-UTR of TAB 2 mRNA in both BMDM and HEK293 cells. However, miR-27a had no effect on the activity of a luciferase reporter that contained the mutant 3′-UTR of TAB 2 mRNA (Figures 7F,G). Additionally, qRT-PCR data demonstrated that BMDM transfected with miR-27a mimic significantly increased the expression levels of miR-27a. On the other hand, BMDMs transfected with miR-27a inhibitor significantly reduced the expression level of miR-27a (Figure 7C). These findings suggest that both IL-10 and TAB 2 are direct targets of miR-27a.

### miR-27a Promotes Antimicrobial Properties of Macrophages and Inhibits Intracellular Survival of MAP

**Figure 8:**
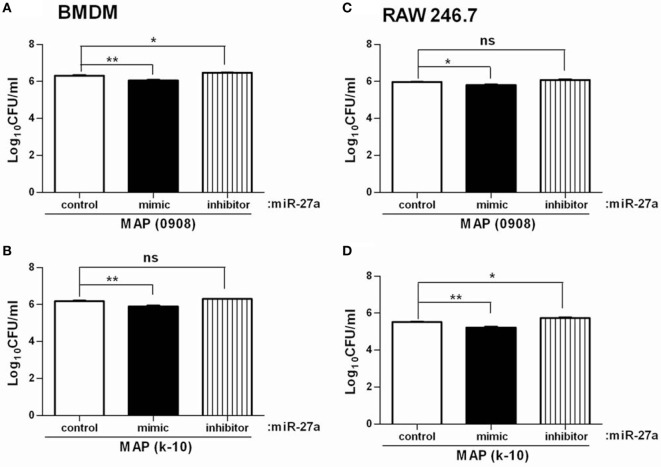
miR-27a promotes antimicrobial properties of macrophages and inhibits intracellular survival of *Mycobacterium avium* subspecies *paratuberculosis* (MAP). Bone marrow-derived macrophages (BMDM) **(A,B)** and RAW 264.7 **(C,D)** cells were transfected with 50 nM miR-27a control, mimic, and inhibitor. Forty-eight hours after transfection cells were infected by MAP (0908 and k-10) strains at multiplicity of infection (MOI) of 20:1 (bacteria/cells) for 18 h. After that, cells were lysed, and intracellular survival of MAP was determined by CFU. Mean ± SD values were obtained from three independent experiments. *p < 0.05 and **p < 0.01.

*Mycobacterium avium* subspecies *paratuberculosis* adopts various strategies for intracellular survival in mononuclear phagocytic cells. The activation of MAPK-p38 signaling pathway and upregulation of IL-10 is one of the important strategies of MAP survival and pathogenesis (58, 59). We already found that miR-27a positively regulates the expression of pro-inflammatory cytokines in MAP-infected macrophages and that overexpression of miR-27a promotes macrophage activation by inhibition of IL-10. We next investigated whether miR-27a affects the intracellular survival of MAP. To test this hypothesis, BMDM cells were transfected with miR-27a control, mimic, and inhibitor. Upregulation of miR-27a reduced the survival of MAP (0908) in BMDM cells, while miR-27a inhibitor counteracted that effect (Figure 8A). Additionally, transfection of RAW264.7 cells with miR-27a mimic showed a clearer difference in the intracellular survival of MAP than cells transfected with miR-27a inhibitor (0908) (Figure 8C). We observed similar results between miR-27a mimic and inhibitor for MAP (k-10)-infected macrophages (Figures 8B,D). Although the difference for total viable bacterial count between the control and miR-27a inhibitor was less evident, there was a clear significant difference between miR-27a mimic and inhibitor group (Figures 8B,C). These results indicate that miR-27a inhibits the intracellular survival of MAP by promoting bactericidal activities of macrophages.

## Discussion

Emerging evidence suggested that miRNAs are key regulators of genes involved in immune cell differentiation, effectors functions, and modulation of the host defense mechanism against pathogenic infection (60, 61). However, the biological role of miRNAs in the specific context of macrophage activation during MAP infection remains poorly understood. Macrophages are not only involved in the elimination of intracellular pathogens but also provide a niche for the survival of MAP organisms. Classically activated macrophages (M1) are pro-inflammatory and are involved in tissue damage in high population numbers (62), while alternatively activated macrophages (M2) are poorly microbicidal (63, 64). In infectious diseases, the control of tissue inflammation might lead to uncontrolled pathogen multiplication (65). Macrophage activation in M1 or M2 must be tightly regulated and can be used as a therapeutic target. Recent studies mentioned that miR-27a and miR-27b play an important role in macrophage polarization by targeting the 3′-UTR of IRF4 (23). In addition, miR-27a is a member of the miR-23a/27a/24-2 gene cluster, promoting the expression of M1 cytokines while reducing the expression of M2 cytokines, suggesting that it plays an important role in the activation and polarization of macrophages (39). Similarly, our results show that miR-27a (miR-27a-3p; a mature form of miR-27a) promotes pro-inflammatory cytokines while inhibiting anti-inflammatory ones and modulating macrophage activation after MAP infection.

We found that the expression of miR-27a was downregulated by MAP (0908 and k-10 strain of MAP) both *in vitro* and *in vivo*. Our results are in accordance with Ma and colleagues findings, where they demonstrated that miR-23a/27a/24-2 was downregulated in mouse peritoneal macrophages stimulated with LPS (39). Similarly, murine cytomegalovirus downregulated the expression of miR-27a in various mouse cell lines and primary macrophages (34).

Several miRNAs are induced by TLR signaling in macrophages and target the 3′-UTR of mRNAs encoding key molecules involved in macrophage polarization (66). TLR2 and TLR4 are important PRRs of phagocytic cells activating MAPK pathway after ligation with the cell wall component Man-LAM and the 19 kDa lipoprotein of MAP (15). Our results revealed that miR-27a is downregulated in BMDM and RAW264.7 cells activated with MAP and TLR2 agonist. Similarly, the study of Xie et al. reported that miR-27a was downregulated in mouse BMDM and human macrophages stimulated by TLR4 and TLR2 agonists (51). Therefore, the regulation of miR-27a appears to be fundamental in keeping macrophage modulation and inflammatory responses.

Farrell and colleagues demonstrated the existence of novel miRNAs in MAP-infected bovine serum and explored the potential of miRNAs as biomarkers for infectious diseases in cattle (37). Our data provide confirmation of the important role of miR-27a in the regulation of IL-10 expression in MAP-infected macrophages. We found that enforced expression of miR-27a suppressed IL-10 protein expression, whereas transfection of cells with an inhibitor of miR-27a resulted in increased IL-10 expression in MAP-infected macrophages. Similarly, it has been shown that miR-27a inhibits the production of IL-10 at both mRNA and protein levels and enhanced the signaling of NF-kB for cytokines production in hypoxia and reperfusion injury (67). Liu and colleagues showed that miR-98 inhibits TLR4-dependent IL-10 production in LPS-stimulated macrophages (68). For the progression of pathogenesis, MAP overexpresses the production of IL-10 that gradually shifts protective Th1-type cell-mediated response to a humoral Th2 response (69). Cho and colleagues reported that miR-24 and miR-27a collectively inhibit the differentiation of CD4 T cells into Th2 cells by targeting IL-4 and GATA3 (33). Similarly, we found that overexpression of miR-27a modulated the immune responses during MAP infection by targeting IL-10 at the 3′-UTR or by inhibiting the regulatory pathway for the expression of IL-10. Previously, it was shown that the regulation of IL-10- and IL-10-dependent MAPK-p38 is required for the control of persistent survival and growth of MAP in infected macrophages (16, 20).

The observed effects on IL-10 expression prompted us to examine whether miR-27a influences the activation of macrophages induced by MAP infection. We demonstrated that the expressions of pro-inflammatory cytokines were upregulated by overexpression of miR-27a, whereas these cytokines were reduced by inhibition of miR-27a by transfection with miR-27a inhibitor. Our work is in line with a previous report showing the effects of miR-23a/27a/24-2 on the expression of pro-inflammatory cytokines in murine macrophages (39). In addition, the expressions of various pro-inflammatory cytokines were suppressed by IL-10 (70). Similarly, the blocking of IL-10 resulted in overexpression of IL-12 and TNF-α in bovine monocyte-derived macrophages infected with MAP or purified protein derivative (or Johnin) (71). Overall, IL-10 is an important anti-inflammatory mediator (72) that blocks the release of various pro-inflammatory cytokines and promotes the survival of MAP in mononuclear phagocytic cells (73). Our data also suggest that mimic treatment of miR-27a inhibits the expression of IL-10, while promoting the regulation of various pro-inflammatory cytokines in murine macrophages results in line with the study reported by Xie and colleagues (51).

Mitogen-activated protein kinase signaling pathways are playing an important role in the regulation of cell death and cell survival depending on the type of stimulus and type of cells (26). The function of the MAPK-p38 branch in the regulation of pro- and anti-inflammatory genes expression is distinct in various cell types (74). In MAP infection, the activation of the MAPK-p38 pathway promotes the production of IL-10 and inhibits antimicrobial activity of macrophages (59). Furthermore, other studies have reported that p38, JNK, and ERK play a dual role in regulating the expression of cytokines, especially IL-12 and IL-10 (75). TAB 2 and its closely related protein, TAB 3, are essential for the activation of TAK1, a major downstream molecule for IkB-kinases and MAPKs including p38 and JNK (76). Previously, it has been shown that TAB 1 and TAB 2 are essential for the survival of LPS-activated macrophages by inhibiting apoptotic cell death *via* preventing reactive oxygen species production (77). Our analysis has revealed that miR-27a significantly inhibited the expression of TAB 2 and TAB 3 and has a putative binding site at the 3′-UTR of TAB 2. These observations prompted us to examine whether miR-27a influences the activation of the MAPK pathway during MAP infection. We found that miR-27a mimic-transfected BMDM cells after infection with MAP (0908) significantly reduced the phosphorylation of p38 and JNK at different times, while no significant difference was recorded for phosphorylated ERK. Previously, it was reported that the inhibition of MAPK-p38 activity promotes the microbicidal ability of bovine monocyte-derived macrophages to eliminate intracellular bacteria (78). In addition, MAP recombinant proteins are involved in the activation of MAPK-p38 in bovine macrophages and resulted in enhanced production of IL-10 (16). We have shown here that miR-27a inhibits IL-10 expression by targeting mRNA at the 3′-UTR of IL-10 as well as inhibition of p38 phosphorylation by targeting TAB 2 mRNA at its 3′-UTR in MAP-infected macrophages. Our results are in line with the finding of Pan and colleagues’ that miR-27a inhibits JNK/p38 expression by targeting MAP2K4 mRNA at its 3′-UTR in human osteosarcoma cells (46).

Macrophages are not only killing mycobacteria by promoting inflammatory mediators but also provide a favorable hiding site for intracellular multiplication and survival of pathogenic mycobacteria (79). Therefore, macrophage activation should be tightly regulated for the development of protective immune responses against mycobacterial infection. An early study reported that miR-27a upregulated the expression of TNF-α in BMDM cells treated with LPS (51), while TNF-α played a crucial role in macrophage activation in killing intracellular mycobacteria (80), indicating that miR-27a might be required in the macrophage-mediated immune defense against infection. We demonstrated that miR-27a enhances pro-inflammatory cytokines, while decreasing IL-10 production and the intracellular survival of MAP, indicating that miR-27a promoted macrophage-mediated bacterial elimination. Similarly, overexpression of miR-27a substantially decreased viral infectivity by repressing the expression of many lipid metabolism-related genes, which are essential for the production of HCV particles. Furthermore, miR-27a enhanced *in vitro* IFN signaling, and patients who expressed high levels of miR-27a in the liver showed a more favorable response to antiviral therapy (81). Forced expression of miR-155 reduced the intracellular growth of mycobacteria by promoting autophagic response in macrophages (82). These studies imply a potential role of miRNAs in the regulation of immune response against invading pathogens. Our results indicate that miR-27a negatively regulates the expression of IL-10 and hence promotes the ability of macrophages to eliminate intracellular MAP.

Emerging evidence suggests the importance of the link between inflammatory mediators and miRNAs during pathogenic infections (83). miR-23a is a member of miR-23a/27a/24-2 cluster, which has been shown to promote macrophage polarization by targeting JAK1 and STAT-6 directly in murine macrophages (39). Previous studies also showed that several pathogens, including mycobacteria, inhibit the classical activation of macrophages and diminished bactericidal activity of macrophages, through the induction of some miRNAs such as miR-125a (84, 85). Similarly, MAP inhibits the expression of multiple miRNAs, which may contribute to MAP evasion strategies from the host defense mechanisms (37, 86, 87). Our findings show that MAP infection inhibits the regulation of miR-27a and that miR-27a is a key mediator of macrophage polarization into M1 phenotype. Therefore, identification of miRNAs related to the dynamic changes of macrophage polarization and understanding their immune regulating functions are important for discussing the molecular basis of disease progression and the development of novel miRNA-targeted therapeutic strategies. Several research groups have explored the role of miRNAs *in vitro* models but few research groups have achieved promising results with miRNAs *in vivo* experiments. Zheng and colleagues reported that the silencing of miR-195 reduced lung and liver injuries and improved the survival of septic mice (88). In addition, the downregulation of miR-328-3p by intratracheal administration of antagomirs of miR-328-3p enhanced bacterial killing when mice were infected with non-typeable *Haemophilus influenza* (89). In the present study, we investigated the role of miR-27a in macrophages infected with MAP *in vitro*, but further studies are required to explore the role of miR-27a in an *in vivo* model of MAP infection.

In conclusion, the interaction between macrophages and MAP remains a challenging subject. However, there is evidence suggesting that macrophages are critical immune-mediating cells playing an important role in the control of MAP infection. Our findings explore the essential role for miR-27a in macrophage activation and MAP elimination and provide important information for the development of therapeutic interventions in paratuberculosis. Although major milestones have been achieved regarding the study of macrophages as an integral part of the innate immune responses against MAP, further molecular research is required to determine whether therapeutic interventions with miR-27a in the modulation of macrophage activation could be a realistic approach for the control of MAP infection in humans and large animals.

## Ethics Statement

All animal experiments were conducted in accordance with the guidelines for the care of laboratory animals, Ministry of Science and Technology People’s Republic of China and approved animal care and use committee (IACUC) protocols at China Agricultural University of Beijing (20110611-01). All other experiments were carried out in accordance with the University Institutional Biosafety Committee (IBC) approved protocol number 20110611-0.

## Author Contributions

TH performed the experiments and wrote the manuscript, and SS and NS helped in critical review and English grammar check. JW and RY helped in experiments related to cell culture and animals infection. YL helped in buying reagents and chemicals for WB experiments. DZ, LY, and XZ gave the conceptual idea for experimental design and critically reviewed the article before final submission.

## Conflict of Interest Statement

All authors read and approved the final manuscript. The authors declare that they have no competing conflict of interests. The reviewer AT and handling editor declared their shared affiliation.

## References

[1] Nielsen SS Toft N. A review of prevalences of paratuberculosis in farmed animals in Europe. Prev Vet Med (2009) 88(1):1–14. 10.1016/j.prevetmed.2008.07.003 18817995

[2] Lombard JE Epidemiology and economics of paratuberculosis. Vet Clin North Am Food Anim Pract (2011) 27:525–35. 10.1016/j.cvfa.2011.07.012 22023831

[3] Lombard JE Gardner IA Jafarzadeh SR Fossler CP Harris B Capsel BR Herd-level prevalence of *Mycobacterium avium* subsp. paratuberculosis infection in United States dairy herds in 2007. Prev Vet Med (2013) 108(2–3):234–8. 10.1016/j.prevetmed.2012.08.006 22979969

[4] Liu X Li J Yang X Wang D Wang J Wu J. The seroprevalence of *Mycobacterium avium subspecies paratuberculosis* in dairy cattle in Xinjiang, Northwest China. Ir Vet J (2017) 70:1–5. 10.1186/s13620-016-0079-0 28070308 PMC5217577

[5] Sorge SS Kurnick S Streevatsan S Detection of *Mycobacterium avium* subspecies *paratuberculosis* in the saliva of dairy cows: a pilot study. Vet Microbiol (2013) 164:383–6. 10.1016/j.vetmic.2013.02.021 23517764

[6] Stevenson K Genetic diversity of *Mycobacterium avium* subspecies *paratuberculosis* and the influence of strain type on infection and pathogenesis: a review. Veterinary research (2015) 46:64. 10.1186/s13567-015-0203-2 PMC4473831 26092160

[7] McNees AL Markesich D Zayyani NR Graham DY *Mycobacterium paratuberculosis* as a cause of Crohn’s disease. Expert Rev Gastroenterol Hepatol (2015) 9(12):1523–34. 10.1586/17474124.2015.1093931 26474349 PMC4894645

[8] Dalziel TK Chronic interstitial enteritis. Br Med J (1913) 2:1068–70.

[9] Liverani E Eleonora S Carla C Paola DM Andrea B *Mycobacterium avium* subspecies *paratuberculosis* in the etiology of Crohn’s disease, cause or epiphenomenon. World J Gastroenterol (2014) 20(36):13060–70. 10.3748/wjg.v20.i36.13060 25278700 PMC4177485

[10] Kuenstner JT Naser S Chamberlin W Borody T Graham DY McNees A. The consensus from the *Mycobacterium avium* ssp. paratuberculosis (MAP) conference 2017. Front Public Health (2017) 5:208. 10.3389/fpubh.2017.00208 29021977 PMC5623710

[11] Thirunavukkarasu S de Silva K Begg DJ Whittington RJ Plain KM. Macrophage polarization in cattle experimentally exposed to *Mycobacterium avium* subsp. *paratuberculosis*. Pathog Dis (2015) 73(9):1–9. 10.1093/femspd/ftv085 26454271 PMC4626599

[12] Astarie DC N’Diaye EN Le Cabec V Rittig MG Prandi J Maridonneau-Parini I. The mannose receptor mediates uptake of pathogenic and nonpathogenic mycobacteria and bypasses bactericidal responses in human macrophages. Infect Immun (1999) 67:469–77. 9916047 10.1128/iai.67.2.469-477.1999 PMC96343

[13] Fratti RA Chua J Deretic V Induction of p38 mitogen-activated protein kinase reduce early endosome autoantigen 1 (EEA1) recruitment to phagosomal membranes. J Biol Chem (2003) 278:46961–7. 10.1074/jbc.M305225200 12963735

[14] Banaiee N Kincaid EZ Buchwald U Jacobs WR Jr Ernst JD Potent inhibition of macrophage responses to IFN-γ by live virulent *Mycobacterium tuberculosis* is independent of mature mycobacterial lipoproteins but dependent on TLR2. J Immunol (2006) 176:3019–27. 10.4049/jimmunol.176.5.3019 16493060

[15] Weiss DJ Souza CD Evanson OA Sanders M Rutherford M. Bovine monocyte TLR2 receptors differentially regulate the intracellular fate of *Mycobacterium avium* subsp. *paratuberculosis* and *Mycobacterium avium* subsp. *avium*. J Leukoc Biol (2008) 83:48–55. 10.1189/jlb.0707490 17913973

[16] Bannantine JP Judith RS Elizabeth L Maria Clara DC Souza CD. *Mycobacterium avium* subspecies *paratuberculosis* recombinant proteins modulate antimycobacterial functions of bovine macrophages. PLoS One (2015) 10(6):e0128966. 10.1371/journal.pone.0128966 26076028 PMC4468122

[17] Cyktor JC Turner J. Interleukin-10 and immunity against prokaryotic and eukaryotic intracellular pathogens. Infect Immun (2011) 79(8):2964–73. 10.1128/IAI.00047-11 21576331 PMC3147550

[18] Wallner FK Hopkins MH Lindvall T Olofsson P Tilevik A Cytokine correlation analysis based on drug perturbation. Cytokine (2016) 90:73–9. 10.1016/j.cyto.2016.10.015 27816795

[19] McCoy CE Sheedy FJ Qualls JE Doyle SL Quinn SR Murray PJ IL-10 inhibits miR-155 induction by toll-like receptors. J Biol Chem (2010) 285(27):20492–8. 10.1074/jbc.M110.102111 20435894 PMC2898307

[20] Nagata R Satoko K Yuu M Xuebo W Tadashi Y Yasuyuki M A specific induction of interleukin-10 by the Map41 recombinant PPE antigen of *Mycobacterium avium* subsp. *paratuberculosis*. Vet Immunol Immunopathol (2010) 135:71–8. 10.1016/j.vetimm.2009.11.002 20018382

[21] Redford PS O’Garra A Murray PJ The role of IL-10 in immune regulation during *M. tuberculosis* infection. Mucosal Immunol (2017) 4(3):261–70. 10.1038/mi.2011.7 21451501

[22] Wong EA Kraus C Reimann KA Flynn JAL The role of IL-10 during early *M. tuberculosis* infection in a non-human primate model. J Immunol (2017) 198(1 Supplement):123.5.

[23] Ahluwalia PK Pandey RK Sehajpal PK Prajapati VK. Perturbed microrna expression by *Mycobacterium tuberculosis* promotes macrophage polarization leading to pro-survival foam cell. Front Immunol (2017) 8:107. 10.3389/fimmu.2017.00107 28228760 PMC5296369

[24] Keshet Y Seger R. The MAP kinase signaling cascades: a system of hundreds of components regulates a diverse array of physiological functions. Methods Mol Biol (2010) 661:3–38. 10.1007/978-1-60761-795-2_1 20811974

[25] Hussain T Shah SZA Zhao Z Sreevatsan S Zhou X. The role of IL-10 in *Mycobacterium avium* subsp. *paratuberculosis* infection. J Cell Commun Signal (2016) 14(29):1–14. 10.1186/s12964-016-0152-z 27905994 PMC5131435

[26] Koul HK Pal M Koul S. Role of p38 MAP kinase signal transduction in solid tumors. Genes Cancer (2013) 4(9–10):342–59. 10.1177/1947601913507951 24349632 PMC3863344

[27] Souza CD. Blocking the mitogen activated protein kinase-p38 pathway is associated with increase expression of nitric oxide synthase and higher production of nitric oxide by bovine macrophages infected with *Mycobacterium avium* subsp *paratuberculosis*. Vet Immunol Immunopathol (2015) 164:1–9. 10.1016/j.vetimm.2015.01.007 25700780

[28] Hoentjen F Sartor RB Ozaki M Jobin C STAT3 regulates NF-kappaB recruit-ment to the IL-12p40 promoter in dendritic cells. Blood (2005) 105:689–96. 10.1182/blood-2004-04-1309 15251981

[29] Guo H Ingolia NT Weissman JS Bartel DP. Mammalian microRNAs predominantly act to decrease target mRNA levels. Nature (2010) 466:835–40. 10.1038/nature09267 20703300 PMC2990499

[30] Drury RE Connor DO Pollard AJ The clinical application of MicroRNAs in infectious disease. Front Immunol (2017) 8:1182 10.3389/fimmu.2017.01182 28993774 PMC5622146

[31] O’Connell RM Rao DS Chaudhuri AA Baltimore D. Physiological and pathological roles for microRNAs in the immune system. Nat Rev Immunol (2010) 10:111–22. 10.1038/nri2708 20098459

[32] Turner ML Schnorfeil FM Brocker T. MicroRNAs regulate dendritic cell differentiation and function. J Immunol (2011) 187(8):3911–7. 10.4049/jimmunol.1101137 21969315

[33] Cho S Wu CJ Yasuda T Cruz LO Khan AA Lin LL miR-23~27~24 clusters control effector T cell differentiation and function. J Exp Med (2016) 213(2):235–49. 10.1084/jem.20150990 26834155 PMC4749926

[34] Buck AJ Perot J Chisholm MA Kumar DA Tuddenham L Cognat V Post-transcriptional regulation of miR-27 in murine cytomegalovirus infection. RNA (2010) 16:307–15. 10.1261/rna.1819210 20047990 PMC2811660

[35] Ji J Zhang J Huang G Qian J Wang X Mei S. Over-expressed microRNA-27a and 27b influence fat accumulation and cell proliferation during rat hepatic stellate cell activation. FEBS Lett (2009) 583(4):759–66. 10.1016/j.febslet.2009.01.034 19185571

[36] Tak H Kim J Jayabalan AK Lee H Kang H Cho GH miR-27 regulates mitochondrial networks by directly targeting the mitochondrial fission factor. Exp Mol Med (2014) 46:1–8. 10.1038/emm.2014.73 25431021 PMC4261914

[37] Farrell D Shaughnessy RG Britton L David E Mac H Bryan M The identification of circulating miRNA in bovine serum and their potential as novel biomarkers of early *Mycobacterium avium* subsp *paratuberculosis* infection. PLoS One (2015) 10(7):e0134310. 10.1371/journal.pone.0134310 26218736 PMC4517789

[38] Shaughnessy RG Farrell D Riepema K Bakker D Stephen V Gordon SV Analysis of biobanked serum from a *Mycobacterium avium* subsp *paratuberculosis* bovine infection model confirms the remarkable stability of circulating miRNA profiles and defines a bovine serum miRNA repertoire. PLoS One (2015) 10(12):e0145089 10.1371/journal.pone.0145089 26675426 PMC4682966

[39] Ma S Liu M Xu Z Li Y Guo H Ge Y A double feedback loop mediated by microRNA-23a/27a/24-2 regulates M1 *versus* M2 macrophage polarization and thus regulates cancer progression. Oncotarget (2016) 7(12):13502–19. 10.18632/oncotarget.6284 26540574 PMC4924657

[40] Meng QL Liu F Yang XY Liu XM Zhang X Zhang C Identification of latent tuberculosis infection-related microRNAs in human U937 macrophages expressing *Mycobacterium tuberculosis* Hsp16.3. BMC Microbiol (2014) 14(37):2–9. 10.1186/1471-2180-14-37 24521422 PMC3925440

[41] Yue R Liu C Barrow P Liu F Cui Y Yang L The isolation and molecular characterization of *Mycobacterium avium* subsp. *paratuberculosis* in Shandong province, China. Gut Pathog (2016) 8(9):2–9. 10.1186/s13099-016-0092-6 27006704 PMC4802609

[42] Thirunavukkarasu S de silva K Whittington RJ Plain KM. In vivo and in vitro expression pattern of toll-like receptors in *Mycobacterium avium* subspecies paratuberculosis infection. Vet Immunol Immunopathol (2013) 15:20–31. 10.1016/j.vetimm.2013.08.008 24054090

[43] El-Etr SH Subbian S Cirillo SLG Cirillo JD Identification of two *Mycobacterium marinum* loci that affect interactions with macrophages. Infection and immunity (2004) 72(12):6902–13. 10.1128/IAI.72.12.6902-6913.2004 15557611 PMC529147

[44] Weaver BK Bohn E Judd BA Gil MP Schreiber RD. ABIN-3: a molecular basis for species divergence in interleukin-10-induced anti-inflammatory actions. Mol Cell Biol (2007) 27:4603–16. 10.1128/MCB.00223-07 17485448 PMC1951479

[45] Srinivasan L Serdar AG Benjamin EH Jessica LM Petros CK Volker B Identification of transcription factor that regulates host cell exit and virulence of *Mycobacterium tuberculosis*. PLoS Pathog (2016) 12(5):e1005652 10.1371/journal.ppat.1005652 27191591 PMC4871555

[46] Pan W Wang H Jianwei R Ye Z MicroRNA-27a promotes proliferation, migration and invasion by targeting MAP2K4 in human osteosarcoma cells. Cell Physiol Biochem (2014) 33:402–12. 10.1159/000356679 24556602

[47] Pooley HB de Silva K Purdie AC Begg DJ Whittington RJ Plain KM A rapid method for quantifying viable *Mycobacterium avium* subsp*. paratuberculosis in* cellular infection assays. Am Soc Microbiol (2016) 112(3–4):203–12. 10.1128/AEM.01668-16 PMC5007765 27371585

[48] Vandesompele J Preter KD Pattyn F Poppe B Roy NV Paepe AD Accurate normalization of real-time quantitative RT-PCR data by geometric averaging of multiple internal control genes. Genome Biol (2002) 3(7):1–12. 10.1186/gb-2002-3-7-research0034 12184808 PMC126239

[49] Gao Y Lin X Shi K Yan Z Wang Z Bovine mammary gene expression profiling during the onset of lactation. PLoS One (2013) 8(8):1–9. 10.1371/journal.pone.0070393 PMC3749150 23990904

[50] Livak KJ Schmittgen TD Analysis of relative gene expression data using real-time quantitative PCR and the 2−ΔΔCT method. Methods (2001) 25(4):402–8. 10.1006/meth.2001.1262 11846609

[51] Xie N Cui H Banerjee S Tan Z Salomao R Fu M miR-27a regulates inflammatory response of macrophages by targeting IL-10. J Immunol (2014) 193:327–34. 10.4049/jimmunol.1400203 24835395 PMC4065847

[52] Shah SZY Zhao D Taglialatela G Khan SH Hussain T Dong H Early minocycline and late FK506 treatment improves survival and alleviates neuroinflammation, neurodegeneration, and behavioral deficits in prion-infected hamsters. Neurotherapeutics (2017) 14(2):463–83. 10.1007/s13311-016-0500-0 28083805 PMC5398981

[53] Huang L Liu Y Wang L Chen R Ge W Lin Z Downregulation of miR-301a suppresses pro-inflammatory cytokines in TLR-triggered macrophages. Immunology (2013) 140(3):314–22. 10.1111/imm.12139 23808420 PMC3800436

[54] Brooks DG Trifilo MJ Edelmann KH Teyton L McGavern DB Oldstone MB. Interleukin-10 determines viral clearance or persistence in vivo. Nat Med (2006) 12:1301–9. 10.1038/nm1492 17041596 PMC2535582

[55] Doyle SL O’Neill LA Toll-like receptors: from the discovery of NF kappa-B to new insights into transcriptional regulations in innate immunity. Biochem Pharmacol (2006) 72(9):1102–13. 10.1016/j.bcp.2006.07.010 16930560

[56] Miranda K Huynh T Tay Y Ang Y Tam W Thomson A A pattern-based method for the identification of MicroRNA binding sites and their corresponding heteroduplexes. Cell (2006) 126:1203–17. 10.1016/j.cell.2006.07.031 16990141

[57] Dweep H Sticht C Pandey P Gretz N miRWalk—database: prediction of possible miRNA binding sites by “walking” the genes of three genomes. J Biomed Inform (2011) 44:839–47. 10.1016/j.jbi.2011.05.002 21605702

[58] Weiss DJ Evanson OA Souza CD Increased expression of interleukin-10 and suppressor of cytokine signaling-3 associated with susceptibility to Johne’s disease. Am J Vet Res (2005) 66:1114–20. 10.2460/ajvr.2005.66.1114 16111147

[59] Souza CD Evanson OA Weiss D Mitogen activated protein kinasep38 pathway is an important component of the anti-inflammatory response in *Mycobacterium avium* subsp. *paratuberculosis* infected bovine monocytes. Microb Pathog (2006) 41:59–66. 10.1016/j.micpath.2006.04.002 16716561

[60] Liston A Linterman M Lu LF. MicroRNA in the adaptive immune system, in sickness and in health. J Clin Immunol (2010) 30:339–46. 10.1007/s10875-010-9378-5 20191314

[61] Lee HM Nguyen DT Lu LF Progress and challenge of microRNA research in immunity. Front Genet (2014) 5:178 10.3389/fgene.2014.00178 24971086 PMC4053854

[62] Redente EF Higgins DM Dwyer-Nield LD Orme IM Gonzalez-Juarrero M Malkinson AM Differential polarization of alveolar macrophages and bone marrow derived monocytes following chemically and pathogen-induced chronic lung inflammation. J Leukoc Biol (2010) 88(1):159–68. 10.1189/jlb.0609378 20360403 PMC2892523

[63] Mosser DM Edwards JP. Exploring the full spectrum of macrophage activation. Nat Rev Immunol (2008) 8:958–69. 10.1038/nri2448 19029990 PMC2724991

[64] Diefenbach A Colonna M Koyasu S. Development, differentiation, and diversity of innate lymphoid cells. Immunity (2014) 41:354–65. 10.1016/j.immuni.2014.09.005 25238093 PMC4171710

[65] Pineros AR Campos LW Fonseca DM Bertolini TB Gembre AF Prado RQ M2 macrophages or IL-33 treatment attenuate ongoing *Mycobacterium tuberculosis* infection. Sci Rep (2017) 7:1–12. 10.1038/srep41240 28128217 PMC5269597

[66] Wu X-Q Dai Y Yang Y Huang C Meng X-M Wu B-M Emerging role of microRNAs in regulating macrophage activation and polarization in immune response and inflammation. J Immunol (2016) 148:237–48. 10.1111/imm.12608 27005899 PMC4913289

[67] Yeh CH Chen TP Wang YC Lin YM Fang SH MicroRNA-27a regulate cardiomyocytic apoptosis during cardioplegia-induced cardiac arrest by targeting interleukin 10yrelated pathways. Shock (2012) 38(6):607–14. 10.1097/SHK.0b013e318271f944 23143062

[68] Liu Y Chen Q Song Y Lai L Wang JL Yu H MicroRNA-98 negatively regulates IL-10 production and endotoxin tolerance in macrophages after LPS stimulation. FEBS Lett (2011) 585(12):1963–8. 10.1016/j.febslet.2011.05.029 21609717

[69] Leite FL Livia BE Bruce P John PB Timothy AR Judith RS. ZAP-70, CTLA-4 and proximal T cell receptor signaling in cows infected with *Mycobacterium avium* subsp. *paratuberculosis*. Vet Immunol Immunopathol (2015) 167:15–21. 10.1016/j.vetimm.2015.06.017 26163934

[70] Del Prete G De Carli M Almerigogna F Giudizi MG Biagiotti R Romagnani S. Human IL-10 is produced by both type 1 helper (Th1) and type 2 helper (Th2) T cell clones and inhibits their antigen-specific proliferation and cytokine production. J Immunol (1993) 150(2):353–60. 8419468

[71] Buza JJ Hikono H Mori Y Nagata R Hirayama S Aodon-geril. Neutralization of interleukin-10 significantly enhances gamma interferon expression in peripheral blood by stimulation with Johnin purified protein derivative and by infection with *Mycobacterium avium* subsp. *paratuberculosis* in experimentally infected cattle with paratuberculosis. Infect Immun (2004) 72:2425–8. 10.1128/IAI.72.4.2425-2428.2004 15039374 PMC375198

[72] Pitt JM Stavropoulos E Redford PS Beebe AM Bancroft GJ Young DB Blockade of IL-10 signaling during bacillus Calmette-Guerin vaccination enhances and sustains Th1, Th17, and innate lymphoid IFN-g and IL-17 responses and increases protection to *Mycobacterium tuberculosis* infection. J Immunol (2012) 189:4079–87. 10.4049/jimmunol.1201061 22972927 PMC3467194

[73] Weiss DJ Evanson OA Souza CD A Critical role of interleukin-10 in the response of bovine macrophages to infection by *Mycobacterium avium* sub *paratuberculosis*. Am J Vet Res (2005) 66:721–6. 10.2460/ajvr.2005.66.721 15900955

[74] Kim C Sano Y Todorova K Carlson BA Arpa L Celada A The kinase p38 alpha serves cell type-specific inflammatory functions in skin injury and coordinates pro- and anti-inflammatory gene expression. Nat Immunol (2008) 9:1019–27. 10.1038/ni.1640 18677317 PMC2587092

[75] Courties G Seiffart V Presumey J Escriou V Scherman D Zwerina J In vivo RNAi-mediated silencing of TAK1 decreases inflammatory Th1 and Th17 cells through targeting of myeloid cells. Blood (2010) 116:3505–16. 10.1182/blood-2010-02-269605 20682854

[76] Xia ZP Sun L Chen X Pineda G Jian X Adhikari A Direct activation of protein kinases by unanchored polyubiquitin chain. Nature (2009) 461:144–99. 10.1038/nature08247 PMC2747300 19675569

[77] Mihaly SR Morioka S Ninomiya-Tsuji J Takaesu G. Activated macrophage survival is coordinated by TAK1 binding proteins. PLoS One (2014) 9(4):e94982. 10.1371/journal.pone.0094982 24736749 PMC3988229

[78] Souza CD Eckstein WC Sreevatsan TM Weiss DJ Mannosylated lipoarabinomannans from *Mycobacterium avium* subsp. *paratuberculosis* alters the inflammatory response by bovine macrophages and suppresses killing of *Mycobacterium avium* subsp. *avium* organisms. PLoS One (2013) 8(9):e75924 10.1371/journal.pone.0075924 24098744 PMC3786972

[79] Liu PT Modlin RL. Human macrophage host defense against *Mycobacterium tuberculosis*. Curr Opin Immunol (2008) 20:371–6. 10.1016/j.coi.2008.05.014 18602003

[80] Bermudez LE Young LS. Tumor necrosis factor, alone or in combination with IL-2, but not IFN-gamma, is associated with macrophage killing of *Mycobacterium avium* complex. J Immunol (1988) 140:3006–13. 2834450

[81] Shirasaki T Honda M Shimakami T Horii R Yamashita T Sakai Y MicroRNA-27a regulates lipid metabolism and inhibits hepatitis C virus replication in human hepatoma cells. J Virol (2013) 87(9):5270–86. 10.1128/JVI.03022-12 23449803 PMC3624309

[82] Wang J Yang K Zhou L Wu M Wu Y Zhu M MicroRNA-155 promotes autophagy to eliminate intracellular mycobacteria by targeting Rheb. PLoS Pathog (2013) 9(10):e1003697 10.1371/journal.ppat.1003697 24130493 PMC3795043

[83] Xu G Zhang Z Xing Y Wei J Ge Z Liu X MicroRNA-149 negatively regulates TLR-triggered inflammatory response in macrophages by targeting MyD88. J Cell Biochem (2014) 115:919–27. 10.1002/jcb.24734 24375488

[84] Banerjee S Cui H Xie N Tan Z Yang S Icyuz M miR-125a-5p regulates differential activation of macrophages and inflammation. J Biol Chem (2013) 288(49):35428–36. 10.1074/jbc.M112.426866 24151079 PMC3853290

[85] Kim JK Yuk JM Kim SY Kim TS Jin HS Yang CS MicroRNA-125a inhibits autophagy activation and antimicrobial responses during mycobacterial infection. J Immunol (2015) 194:1–11. 10.4049/jimmunol.1402557 25917095

[86] Malvisi M Palazzo F Morandi N Lazzari B Williams JL Pagnacco G Responses of bovine innate immunity to *Mycobacterium avium* subsp. *paratuberculosis* infection revealed by changes in gene expression and levels of MicroRNA. PLoS One (2016) 11(10):e0164461. 10.1371/journal.pone.0164461 27760169 PMC5070780

[87] Sharma N Kumawat KL Rastogi M Basu A Singh SK Japanese encephalitis virus exploit the microRNA-432 to regulate the expression of suppressor of cytokine signaling (SOCS) 5. Sci Rep (2016) 6:1–12. 10.9734/JSRR/2016/17929 27282499 PMC4901348

[88] Zheng D Yu Y Li M Wang G Chen R Fan GC Inhibition of microRNA 195 prevents apoptosis and multiple-organ injury in mouse models of sepsis. J Infect Dis (2016) 213:1661–70. 10.1093/infdis/jiv760 26704614 PMC4837905

[89] Hock LT Kaiko GE Plank M Li J Maltby S Essilfie AT Antagonism of miR-328 increases the antimicrobial function of macrophages and neutrophils and rapid clearance of non-typeable *Haemophilus influenzae* (NTHi) from infected lung. PLoS Pathog (2015) 11(4):1–21. 10.1371/journal.ppat.1004549 PMC4404141 25894560

