# Supplementary material for: MicroRNA 27a-3p Regulates Antimicrobial Responses of Murine Macrophages Infected by Mycobacterium avium subspecies paratuberculosis by Targeting Interleukin-10 and TGF-β-Activated Protein Kinase 1 Binding Protein 2
Source: Front Immunol. 2018 Jan 11;8:1915. doi: 10.3389/fimmu.2017.01915 (PMC5768609; doi:10.3389/fimmu.2017.01915)
Supplement: Supplementary file 1 [file Data_Sheet_1.docx]

**Figure Legends**

**Figure-1**. **MAP infection decreases miR-27a expression in macrophages and mice**

(A and B) BMDM and (D and E) RAW264.7cells were infected with MAP (k-10 and 0908) for the indicated time period, and miR-27a expression was subsequently evaluated by using qRT-PCR. (C) RAW264.7 and (F) BMDM were stimulated with 1 µg/ml Pam3Cys-Ser-(Lys)4 (PAM TLR1/2 agonist) for the indicated time period, and miR-27a expression was examined using qRT-PCR. (G, H and I) the expression levels of miR-27a were measured in the intestine (ilium) (G), spleen (H) and liver (I) of negative control or MAP (0908) infected C57BL/6 mice by qRT-PCR analysis. All data above represent mean ±SD for three independent experiments. **p*<0.05, ***p*<0.001

**Figure-2.** **miR-27a attenuates the regulation of IL-10 in macrophages infected by MAP.** (A and B) BMDM, (D) RAW264.7 cells were transfected with 50nM control mimics or miR-27a mimics. After 48 hours cells were infected with MAP (0908 or K-10) strain for 6 and 18 hours. The mRNA and protein levels of anti-inflammatory cytokine, IL-10 were calculated by using qRT-PCR and ELISA. (E and F) BMDM, (H) RAW264.7 cells were transfected with 50nM control inhibitors or miR-27a inhibitors. After 48 hours cells were infected with MAP (0908 or K-10) strain for 6 and 18 hours. The mRNA and protein levels of anti-inflammatory cytokine, IL-10 were determined by qRT-PCR and ELISA. (C and G) BMDMs were transfected with miR-27a control, mimic (C), or inhibitor (G) and then infected with MAP. The expression levels of miR-27a were determined by qRT-PCR analysis. Statistical analysis was carried out by using one-way ANOVA followed by Bonferroni’s multiple comparison tests. **p*<0.05, ***p*<0.001, ****p*<0.001

**Figure-3.** **miR-27a upregulation improves macrophage activation in response to MAP infection.** (A-D) BMDM cells were transfected with 50nM miR-27a control or miR-27a mimic. After 48 hours cells were infected by MAP (0908 or k-10) strain for 6 hours. The mRNA and protein levels of pro-inflammatory cytokines, IL-1β, IL-6, IL-12, IFN-β and TNF-α were determined by qRT-PCR (A and C) and IL-6, IL-12, and TNF-α were determined by ELISA (B and D). (E-H) RAW264.7 cells were transfected with 50nM miR-27a control or miR-27a mimic. After 48 hours cells were infected by MAP (0908 or k-10) strain for 6 hours. The mRNA and protein levels of pro-inflammatory cytokines, IL-1β, IL-6, IL-12, IFN-β and TNF-α were determined by qRT-PCR (E and G) and IL-6, IL-12, and TNF-α were determined by ELISA (F and H). Data represent the mean ±SD from three independent experiments. **p*<0.05, ***p*<0.001, ****p*<0.001

**Figure-4.** **Downregulation of miR-27a inhibits inflammatory responses of MAP infected macrophages.** (A-D) BMDM cells were transfected with 50nM control inhibitors or miR-27a inhibitors. After 48 hours cells were infected by MAP (0908 or k-10) strain for 6 and 18 hours. The mRNA and protein levels of pro-inflammatory cytokines, IL-1β, IL-6, IL-12, IFN-β and TNF-α were determined by qRT-PCR (A and C) and IL-6, IL-12, and TNF-α were determined by ELISA (B and D). (E-H) RAW264.7 cells were transfected with 50nM control inhibitors or miR-27a inhibitors. After 48 hours cells were infected by MAP (0908 or k-10) strain for 6 and 18 hours. The mRNA and protein levels of pro-inflammatory cytokines, IL-1β, IL-6, IL-12, IFN-β and TNF-α were determined by (E and G) qRT-PCR and IL-6, IL-12, and TNF-α were determined by (F and H) ELISA. Similar results were observed in three independent experiments. **p*<0.05, ***p*<0.01, ****p*<0.001

**Figure-5**.**miR-27a modulates MAPK signaling cascades by targeting TAB2 and TAB3 in MAP infected macrophages.** (A) BMDM cells were transfected with 50 nM miR-27a control, mimic and inhibitor for 48 hours and then infected with MAP (0908) at MOI of 20 for 30 minutes, 6 h and 18h. The expression levels of TAB2, TAB3, p-P38, p-JNK and p-ERK were detected by western-blot. (B-F) Western-blot analysis of protein levels of TAB2 (B) , TAB3 (C) , p-P38 (D) , p-JNK (E) s, and p-ERK (F) , normalized to GAPDH after transfection with miR-27a control, mimic and inhibitor for the indicated times. Data represent the mean ±SD from three independent experiments. **p*<0.05, ***p*<0.001, ****p*<0.001

**Figure-6.** **IL-10 and TAB2 are direct targets of miR-27a.** (A) The mature sequences of miR-27a/b and the conserved sequences of the 3′ UTR of IL-10 and TAB2 from various species are illustrated. The seed sites of miR-27a/b and its binding sites at the 3-UTR of IL-10 and TAB2 are shown in *blue* and by the *red* *color*, respectively. (B) Schematic illustration of the miR-27a targeting site at 3′ UTR of the mouse IL-10 and TAB2 gene. The created mutation is represented in green color. (C) BMDM cells were transfected with 50 nM miR-27a control, mimic and inhibitor. Forty eight hours after transfection cell were lysed, and the expression levels of miR-27a were determined by real-time PCR assay. Data represent the mean ±SD from two independent experiments. (D) BMDM cells were transfected with 50nM with miR-27a control, miR-27a mimic, and a wild type (IL-10 WT) or mutant IL-10 3′UTR (IL-10 Mut) and (F) wild type (TAB2 WT) or mutant TAB2 3′UTR (TAB2 Mut) luciferase reporter plasmid, and the luciferase activity of the BMDM cells were assessed at 24 h after transfection. (E) HEK-293 cells were transfected with 50nM with miR-27a control, miR-27a mimic, and a wild type (IL-10 WT) or mutant IL-10 3′UTR (IL-10 Mut) and (G) wild type (TAB2 WT) or mutant TAB2 3′UTR (TAB2 Mut) luciferase reporter plasmid, and the luciferase activity of the HEK-293 cells were assessed at 24 h after transfection. Similar results were observed in three independent experiments. **p*<0.05, ***p*<0.01, ****p*<0.001

**Figure-7.** **miR-27a promotes antimicrobial properties of macrophages and inhibits intracellular survival of MAP.** (A and B) BMDM and (C and D) RAW 264.7 cells were transfected with 50 nM miR-27a control, mimic and inhibitor. Forty eight hours after transfection cell were infected by MAP (0908 and k-10) strains at MOI of 20 for 18 hours, cells were lysed, and intracellular survival of MAP was determined by CFU. Mean ±SD were obtained from three independent experiments. **p*<0.05, ***p*<0.01

**Figure 8: Schematic diagram for the role of miR-27a in MAP mediated signaling pathways in macrophage.** MAPK pathway is triggered by TLRs, TLR2/4 is more critical in signaling through various adopter proteins. A complex of protein integrations are involved in the downsignaling cascades consisting of TGF*β*-activated protein kinase 1 (TAK1), TAK binding protein1 (TAB1) and TAB2 or TAB3. This interaction induces phosphorylation of TAK1 which results in the activation of the MAPK-p38 (mitogen-associated protein kinases). This leads to the activation and nuclear translocation of transcription factors, which plays a critical role in the expression of pro-inflammatory and anti-inflammatory cytokines such as IL-10. MAP predominantly induces an immunosuppressive cytokine IL-10 which subverts protective immune responses and promotes MAP growth and survival. MAP downregulates the expression of miR-27a, while miR27a inhibit the activation of MAPK-p38 signaling pathway by targeting TAB2. Furthermore, miR-27a negatively regulates the expression of IL-10 by directly targeting IL-10 mRNA at its 3 ′UTR.

**Supplementary Figure 1. Cell infectivity assay.** (A and B) RAW264.7 cells were cultured on small cover glasses in 24 well plates infected by MOI of 5, 10 and 20 with live and Killed MAP (0908) for three hours then washed with warm PBS and again incubated with fresh medium. (A) Twenty four hours post-infection cells with MOI of 10 and 20 were washed with PBS fixed and stained for acid fast bacilli and observed under a microscope. (B) The number of infected cells was calculated by counting the infected cells out of at least 100 macrophages in different microscopic fields. Macrophages were scored as percentage of macrophages with acid-fast bacilli (AFB), and for the estimated number of intracellular AFB. Data represent the mean ±SD from three independent experiments. **p*<0.05, ***p*<0.01

**Supplementary Figure 2. Upregulation of miR-27a maintains the expression of pro-inflammatory cytokines in macrophages infected by MAP**. (A and B) BMDM, (C) RAW 264.7 cells were transfected with 50nM miR-27a control or miR-27a mimic. After 48 hours cells were infected by MAP (0908 or k-10) strain for 18 hours. The mRNA and protein levels of pro-inflammatory cytokines, IL-6, IL-12, and TNF-α were determined by qRT-PCR (A) and IL-6, IL-12, and TNF-α and by ELISA (B and C). Data represent the mean ±SD from three independent experiments. **p*<0.05, ***p*<0.01

**Supplementary Figure 3. Downregulation of miR-27a inhibits pro-inflammatory cytokines production in MAP infected macrophages.** (A and B) BMDM, (C) RAW 264.7 cells were transfected with 50nM control inhibitors or miR-27a inhibitors. After 48 hours cells were infected by MAP (0908 or k-10) strain for 18 hours. The mRNA and protein levels of pro-inflammatory cytokines, IL-6, IL-12, and TNF-α were determined by qRT-PCR (A) and IL-6, IL-12, and TNF-α and ELISA (B and C). Data represent the mean ±SD from three independent experiments. **p*<0.05, ***p*<0.01

**Supplementary Figure 4. Cell viability assay and Schematic illustration of miR-27a and miR-27b in various species.** (A) BMDM, (B) RAW264.7 cells were transfected with 50nM/ml control mimic, mimic, control inhibitors or miR-27a inhibitors. After 48 hours 20ul of MTS (promega) reagent was added in each well and incubated at 37°C for 3 hours in a humidified, 5% CO2 atmosphere. Optical density (OD) was quantified by measuring the absorbance at 490 nm wavelength with an ELISA plate reader. (C) The mature sequences of miR-27a/b from various species are illustrated. The seed sites of miR-27a/b that bind at 3′ UTR of mRNAs are shown in *red* and the difference of one nucleotide from miR-27a to miR-27b are shown by *blue* and *purple color*, respectively.
