# Supplementary figures and images for: MicroRNA 27a-3p Regulates Antimicrobial Responses of Murine Macrophages Infected by Mycobacterium avium subspecies paratuberculosis by Targeting Interleukin-10 and TGF-β-Activated Protein Kinase 1 Binding Protein 2
Source: Front Immunol. 2018 Jan 11;8:1915. doi: 10.3389/fimmu.2017.01915 (PMC5768609; doi:10.3389/fimmu.2017.01915)

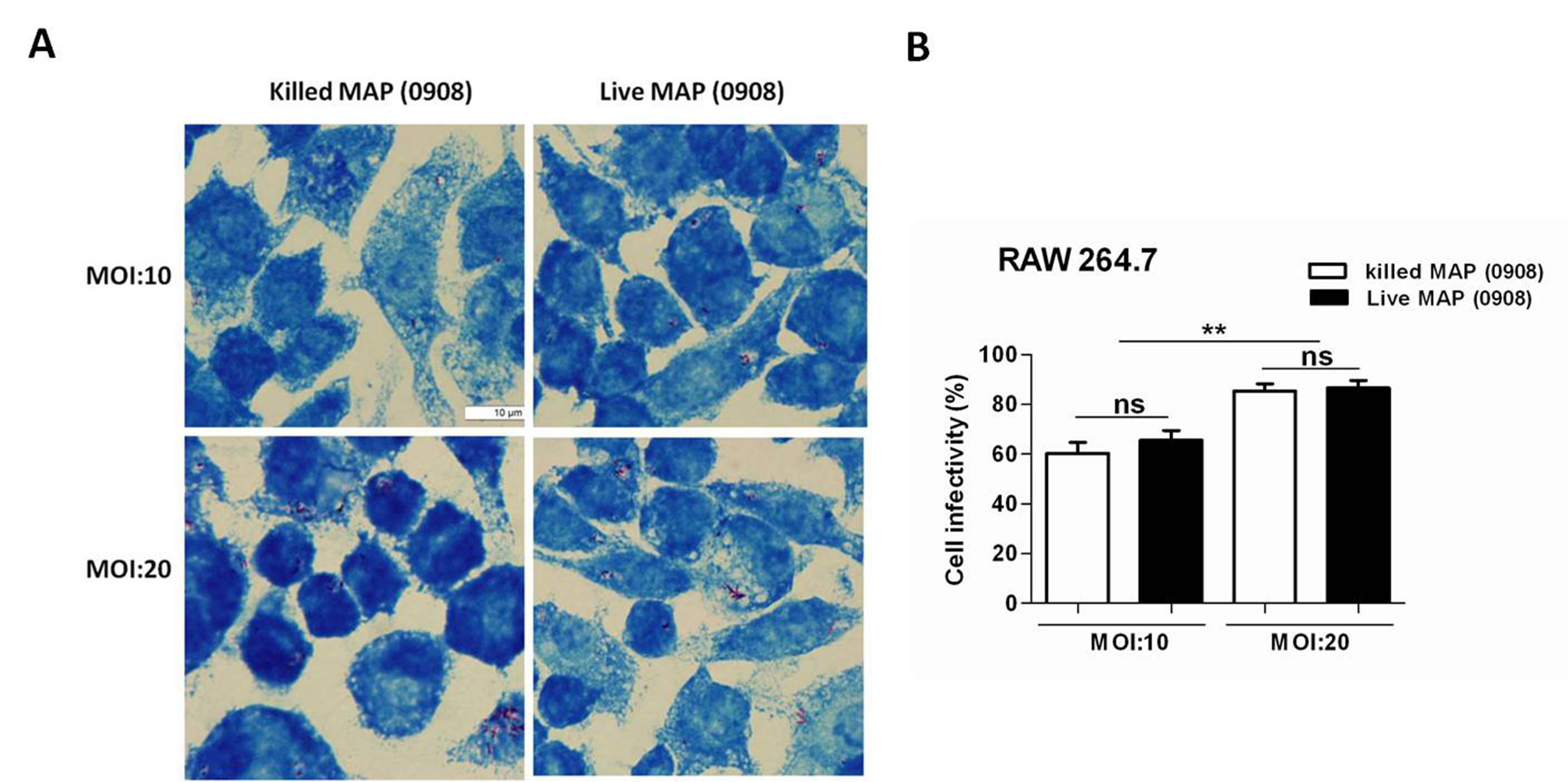

Supplement: Supplementary file 2 [file Image_1.tif]

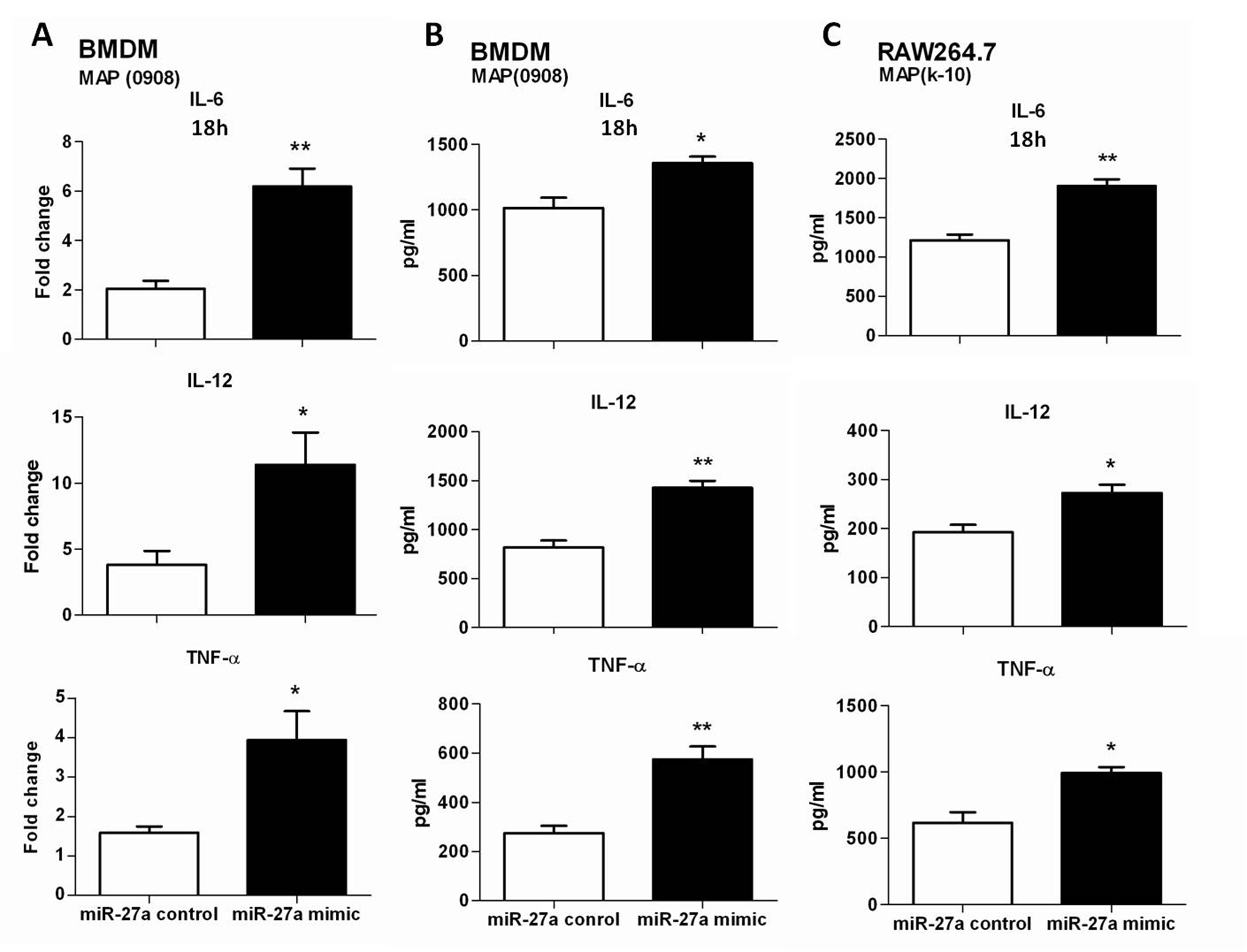

Supplement: Supplementary file 3 [file Image_2.tif]

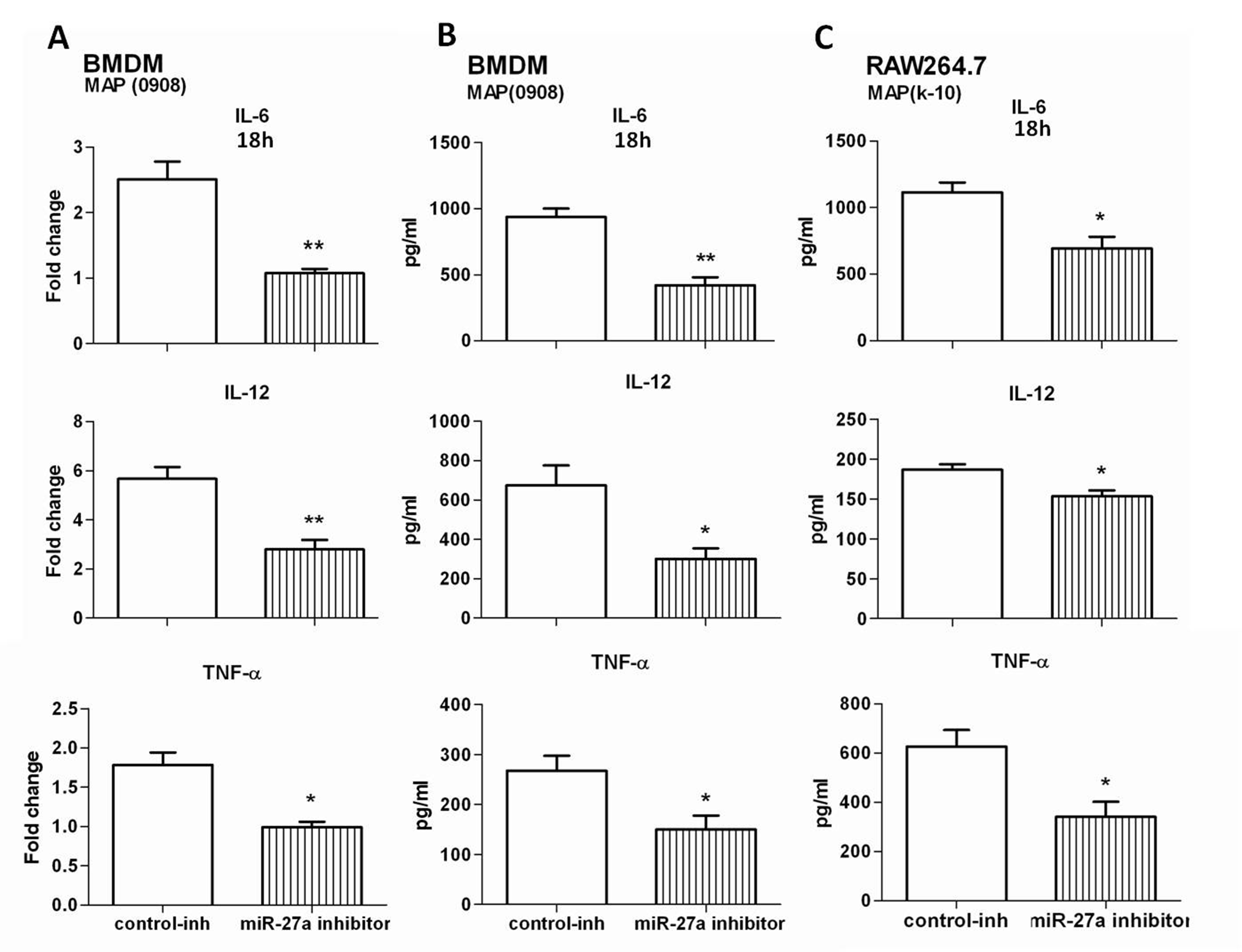

Supplement: Supplementary file 4 [file Image_3.tif]

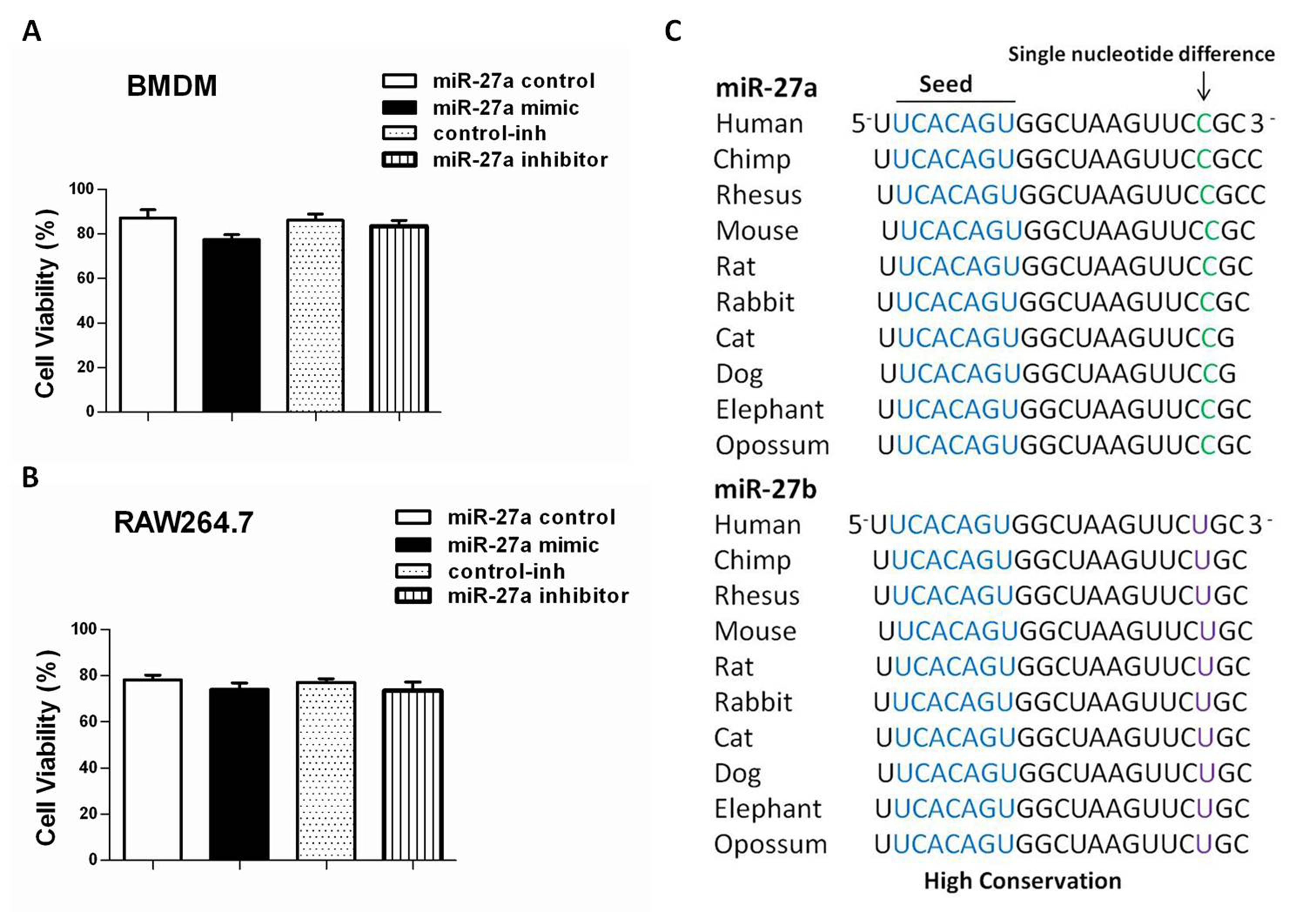

Supplement: Supplementary file 5 [file Image_4.tif]
